# Mechanisms for Cognitive Impairment in Epilepsy: Moving Beyond Seizures

**DOI:** 10.3389/fneur.2022.878991

**Published:** 2022-05-12

**Authors:** Mohamed R. Khalife, Rod C. Scott, Amanda E. Hernan

**Affiliations:** ^1^Division of Neuroscience, Nemours Children's Health, Wilmington, DE, United States; ^2^Psychological and Brain Sciences, University of Delaware, Newark, DE, United States; ^3^Institute of Child Health, Neurosciences Unit University College London, London, United Kingdom

**Keywords:** epilepsy, cognition, neural coding, information processing, place cells, population coding, phase precession

## Abstract

There has been a major emphasis on defining the role of seizures in the causation of cognitive impairments like memory deficits in epilepsy. Here we focus on an alternative hypothesis behind these deficits, emphasizing the mechanisms of information processing underlying healthy cognition characterized as rate, temporal and population coding. We discuss the role of the underlying etiology of epilepsy in altering neural networks thereby leading to both the propensity for seizures and the associated cognitive impairments. In addition, we address potential treatments that can recover the network function in the context of a diseased brain, thereby improving both seizure and cognitive outcomes simultaneously. This review shows the importance of moving beyond seizures and approaching the deficits from a system-level perspective with the guidance of network neuroscience.

## Introduction

Epilepsy is a disorder of the brain characterized by an enduring predisposition to generate seizures and by the neurobiological, cognitive, psychological, and social consequences of this condition (1). Although seizures are an important part of the definition, the associated cognitive and behavioral impairments and learning and memory problems are also important determinants of quality of life (2, 3). The quality of life is highly affected in patients with epilepsy especially if they are in adolescence, affecting the patient's self-esteem and sense of coherence (4). Self-esteem is a main contributor to psychosocial wellbeing, personal reflection, and positive attitude (5), and coherence is the ability to recognize stressors as manageable and solvable (4, 6). In a 5-year follow up study, it was shown that sense of coherence decreased in adolescents with epilepsy. Adolescents who still had seizures showed a greater decrease compared to seizure-free teens, with no effect of seizure frequency on sense of coherence (4). Self-esteem was also decreased in adolescents with epilepsy, however, self-esteem was affected by the seizure frequency where higher seizure frequency was associated with lower self-esteem (4). This indicates that psychosocial wellbeing is affected in adolescents with epilepsy seen by the decrease of both self-esteem and sense of coherence.

Gauffin et al. (7) examined the experience of living with epilepsy and cognitive decline. Their study found out that cognitive decline is persistently present in adults with intractable epilepsy and living with epilepsy and cognitive deficits affected education, employment, self-esteem, social life, and future plans (7). The cognitive deficits are seen in both focal and generalized seizures. Focal seizures occur in a lateralized network in contrast to generalized seizures occurring in a widespread network encompassing both hemispheres (8), however similar cognitive impairments are seen in both epilepsies (9). Patients with focal epilepsy experience various cognitive impairments such as language abnormalities, executive dysfunction, attention deficit and long-term episodic and semantic memory deficits (9–11). Patients with generalized epilepsy experience the same cognitive impairments in addition to acquired knowledge deficits and long-term information processing and retrieval impairment (9, 12, 13).

**Figure 1:**
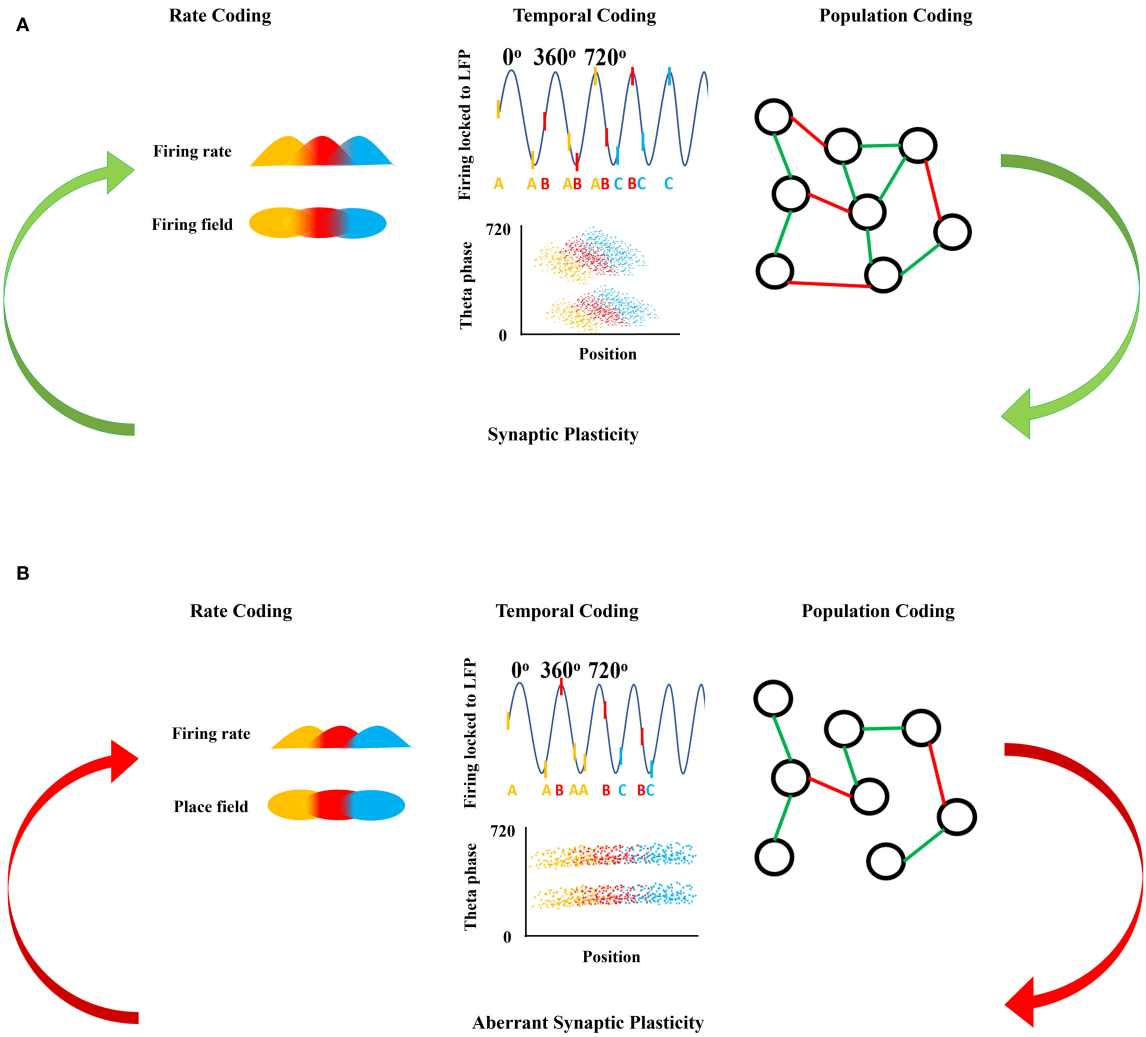
Neuronal firing mechanisms and information processing. **(A)** Shows the normal information processing and synaptic plasticity feedback loop based on the neural coding mechanisms. Rate coding is shown as the firing rate in time (top) with respect to each firing field in space (bottom) coded by the same color. Temporal coding shows the action potentials as raster ticks riding on the LFP with respect to theta oscillation indicating theta phase precession with each successive peak of theta, with action potentials also represented as a letter at the bottom of the LFP representing firing from the same neuron over time (top). Over many LFP cycles, theta phase precession can be seen in the downward slope of the clouds of dots in the bottom panel, each representing an action potential from three representative neurons show in the top panel. Population coding shows the connections between neurons forming a population network, green representing an excitatory connection between two neurons and red representing an inhibitory connection between two neurons. Synaptic plasticity refines and is refined by these firing mechanisms. **(B)** Shows the disrupted information processing and synaptic plasticity feedback loop based on the neural coding mechanisms in epileptic brain. In epilepsy, rate coding is disrupted shown here as a decreased firing rate in time with respect to each firing field in space, with decreased overlap in the firing and place fields from each of the three color-coded neurons. Temporal coding is also altered; firing of the colored neurons with respect to theta oscillation is disorganized and there is an absence of theta precession as shown by a flat relationship between the clouds of action potentials from each of the three colored neurons. Population coding shows fewer connections between neurons forming a smaller population network, with potentially different proportions of positive (green) and negative (red) connections in the epileptic brain compared to controls in **(A)**. Aberrant synaptic plasticity occurs as a result of the aberrant firing dynamics.

A common pathophysiological argument for the mechanisms of the relationship between epilepsy and cognition is that the seizure and epileptiform discharges in the EEG directly injure neural networks that are the normal substrate for cognitive function. An alternative hypothesis is that the relationship is indirect; both the seizures and the additional morbidities arise from neural networks that have been disrupted by the etiology of the epilepsy e.g., single gene disorders, malformations of cortical development, or traumatic brain injury. Our starting position is that the action potential is the fundamental unit of information processing in the brain, and that sequences of action potential firing in neuronal populations over time are therefore considered to be mechanisms of cognition, as cognitive function is explicitly about information processing (14, 15). Our goal in the current article is to explore the latter hypothesis in detail, starting at the systems level physiology of brain function and relating this exploration to our understanding of epilepsy. We will describe these system level mechanisms in physiology (Figure 1A) and discuss how these mechanisms are changed or altered in brain diseases associated with epilepsy (Figure 1B), especially in the hippocampus and neocortex, which are important structures involved in memory.

## Rate Coding

The firing rate of neurons in the brain is a mechanism of information transfer (15). For example, in motor neurons, the degree of muscle flexion depends on the number of action potentials per unit time (14). Tactile and texture perception in rodents is also, at least in part, a function of rate coding (16). In hippocampus and entorhinal cortex, rate coding is illustrated by place, time, and grid cells, respectively (17, 18).

Place cells are hippocampal pyramidal cells that fire when an animal visits a specific region of the environment: the cell's “place field” (19, 20). In any given environment, place fields cover the entire space to create a hippocampal cognitive map that is a representation of that space (19, 20). Repeated recordings of place cell populations have shown that the same cells are activated whenever the animal visits the same region in physical space, which suggests that the cells' representation is held in the network once the animal explores that specific field (21). This representation allows recollection of specific spaces and accurate navigation through the environment. This is further supported by the observation that lesioning the hippocampus results in loss of spatial memory (22). Similarly to place cells, time cells in the hippocampus fire at specific times in a task (e.g., the beginning, middle, or end) called time fields (23). These cells can be time locked to an external stimulus, like a tone, or intrinsically by a neural circuit or an oscillation (14, 15).

Grid cells are entorhinal cortical cells that provide activity-based maps of speed and direction in a certain environment (24). Grid cells fire in different locations in an environment forming a triangular grid. Recording different cells at the same location indicates that these cells have the same orientation to the environment (24, 25). It is important to note that the orientation of the grid relative to the environment is dependent on the hippocampal place cell map (20, 25, 26). Interestingly, the map formed by the grid cells is based on external cues, however the formed map persists even in the absence of these cues (24). Together with the place cell map, the grid cell map is believed to be part of the greater hippocampal cognitive map (20). Given what we know from work on place and grid cells, further experiments have shown that firing rate variations in CA3 place cells depended on signals from the lateral entorhinal cortex (24, 27). Lesioning the lateral entorhinal cortex impairs the hippocampal rate remapping upon changing the configuration of the environment (24, 27) suggesting that inputs from the entorhinal cortex are important for hippocampal rate coding in the formation of the spatial memory and cognitive map.

## Population Coding

Rate coding as measured in individual neurons is essential for information processing. However, neurons are functionally connected into a network and interactions between the neurons is also critically important (15, 28). This is known as population coding. Population coding increases robustness of network function and minimizes the effects of noise carried by an individual neuron, ensuring that the signal and processing times are not affected (15). For example, damage to one cell will not have a devastating effect on the information being carried since it is carried by many cells (28). Population coding is common in the nervous system and is illustrated in mammalian visual pathways (29), primary motor cortex activity in cats and monkeys (30), owl auditory cortex, cricket nervous system (31), and mice visual cortex (32–34). Neurons in the visual cortex in cats and monkeys and the auditory cortex in owls have shown the ability to synchronize their firing on a few milliseconds time scale through time cells (14, 35). Synchrony seen in the visual cortex of cats and monkeys takes place when the neurons are activated by one stimulus (35), and this synchrony is lost when the neurons are activated by two independent stimuli. Once two neurons synchronize to represent a certain stimulus, these two neurons always synchronize to represent the same stimulus and will desynchronize when representing two independent stimuli (14).

Population coding influences information processing in mice visual systems. Whole cell recordings in layers 2/3 (L2/3) of awake mice have shown that the excitation/inhibition ratio changes based on the visual stimulus (33). Different studies revealed that patterns of excitation and inhibition are generated in response to various visual stimuli (36, 37). For example, as the stimulus contrast or size increases, the excitation/inhibition ratio decreases. This change in ratio is important for tuning and sharpening of the information processing in the visual system (38) and is thought to be controlled by the somatostatin (SOM) neurons and parvalbumin (PV)-expressing cells. SOM neuron suppression was able to enhance the excitation and inhibition for size tuning, indicating that contrast and size in the visual system in mice depends on excitation/inhibition ratio and tuning of total synaptic input (33). PV cell manipulation was shown to modulate L2/3 pyramidal cells spikes in response to visual stimuli without affecting the cells tuning properties suggesting that PV cells create a connection between certain neuron types and specific computations during sensory processing (32, 39). Furthermore, the excitation/inhibition ratio for the same set of stimuli differs between anesthetized and awake mice and even differs between various behavioral states (33). This change in the ratio is influenced by the recruitment of SOM and vasoactive intestinal peptide-expressing (VIP) inhibitory cells in V1 in both wakefulness and alertness for instance (40–42). The Stabilized Supralinear Network (SSN) model proposes that cortical dynamics can change the excitation/inhibition ratio based on single neurons input/output supralinear relationships, strong recurrent excitation, and feedback inhibition (33, 43). This suggests that the dynamic change of excitation/inhibition ratio depends on the dynamic connections between different cortical regions and the feedback loops between the recruited regions. Further support to this suggestion was seen in experiments investigating the rhythmic activity across cortical layers. Population of excitatory neurons in the mouse's primary visual cortex expressed gamma band oscillation following layer-specific optogenetic stimulation (34). Importantly, rhythm-generating circuits in each layer were able to provide layer-specific excitation/inhibition balances hence influencing the information flow between cortical layers (34). These studies provide evidence that neuronal populations can be recruited through precise synchrony contributing to information processing at a system level.

Notably, place cells in the hippocampus are an excellent example of not only rate coding, but also population dynamics. We previously mentioned that the place cells fire whenever the animal is in a specific place field and these cells are distributed within the hippocampus in a way that covers the whole environment the animal occupies. This distribution within the hippocampus will cause a certain degree of overlap between the place fields, thus a population of cells will respond when the animal goes into the field rather than an individual cell since the cells in the hippocampus are receiving multiple sensory inputs to encode a multidimensional map (44).

Another interesting example of population coding is pattern separation. Pattern separation depends on the discrimination between two closely related places, episodes, or spatial configurations based on experience and can influence successful memory encoding. Pattern separation involves different brain regions where experiences are represented by neural populations. Notably, if the same population or neural pattern established during encoding is activated during retrieval, it can lead to a successful memory retrieval (45). For instance, both the posterior occipitotemporal cortex (OTC) and the hippocampus were recorded while participants performed item recognition tasks. Upon retrieval, both regions showed encoding-specific high frequency activity (HFA) where the strength of this activity was associated with enhanced retrieval, however the discrimination between similar items required a hippocampal activity (45) as the pattern separation mechanism is based on orthogonalizing similar input during encoding thus enabling the distinguishing between highly similar memories with minimal interference (46). Animal and human studies have shown that dentate gyrus (DG) and its projection to CA3 underlie the pattern separation process (47–52).

A final relevant example of population coding is working memory in the prefrontal cortex. Working memory is the temporary maintenance of information involving specialized components of cognition that allows retaining immediate past-experience, supporting new knowledge acquisition, solving problems, reasoning, and planning (53). Early models of working memory suggested that persistent firing activity of the neurons in the prefrontal cortex (PFC) throughout the delay phase of the working memory task was required to maintain information in working memory, however, due to the heterogeneity of neurons within the PFC, recent work has shown that the persistent activity of PFC can be weak or absent (54–56). This was seen during the delay phase of an image-sequence matching task in monkeys and humans. In this task, spiking activity in the PFC decreased during the delay phase of the task in monkeys, and BOLD signal on fMRI decreased during the delay phase in humans performing the task, thus challenging the persistent firing working memory model (57, 58). However, the spiking activity and the BOLD signal re-emerge during image presentation and testing period, indicating that, despite a decrease in firing rate throughout the task, the working memory information is maintained in the collective synaptic weights of populations of neurons in the PFC. The heterogeneity of the neurons allows different neurons to signal during different task events as well. For example, parvalbumin-positive neurons will respond to sensory cues and trial outcomes while somatostatin-positive neurons will respond to a motor action like licking (59). These neurons can fire together in working memory maintenance through population coding when a robust mnemonic stimulus is present (60) as seen in monkeys performing oculomotor delayed response and vibrotactile delayed discrimination, which are working memory tasks, while recording single neurons of lateral PFC (60).

## Temporal Coding and Oscillatory Firing

Oscillatory activity is divided into frequency bands as described in Table 1: infra-slow oscillations (0.5–1 Hz), delta (1.5–4 Hz), theta (4–8, 10 Hz), alpha (8, 10–12 Hz), beta (15–30 Hz), gamma (30–80 Hz), in addition to fast (80–200 Hz), and ultra-fast (200–600 Hz) ripples (61). Each band is thought to be related to specific aspects of cognition (Table 1). Both *in vivo* and *in vitro* experiments suggest that synaptic inhibition plays a role in generating neuronal oscillations through two different mechanisms, either through interneuron network activity or reciprocal excitatory-inhibitory loops (62). Theta, as previously discussed, and gamma are two important readouts of the hippocampal function and function of connected regions.

**Table 1:** Frequency bands and cognitive processes.

**Bands**	**Frequency (Hz)**	**Cognitive processes**
Infra-slow	0.5–1	Show resting state networks (RSNs) in awake human subjects
Delta	1.5–4	Anticipation and predictive coding
Theta	4–8 (humans) 6–10 (rodents)	Spatial navigation, working memory, and temporal coding
Alpha	8, 10–12	Suppression and selection of attention
Beta	15–30	Involved in consciousness, logical/active thinking, focus, and stress
Gamma	30–80	Readout of information transfer from CA3 to CA1 for hippocampal memory retrieval. Show the temporal organization of movement sequences, memory encoding and formation, sensory processing and planned trajectories underlying spatial navigation.
Fast ripples	80–200	Show synchronous inhibitory postsynaptic potentials (IPSP) generated by interneuronal cell subpopulations
Ultra-fast ripples	200–600	Show synchronous inhibitory postsynaptic potentials (IPSP) generated by interneuronal cell subpopulations

Theta rhythm indicates a network that is actively involved in spatial navigation, working memory, and temporal coding (63, 64). It is important to note that theta oscillations and phase-locked neuron discharges with respect to theta oscillations are seen in theta non-generating regions like entorhinal cortex, perirhinal cortex, cingulate cortex, subicular complex, and amygdala (63, 65–67). Lesion or inactivation of the medial septum-diagonal band of Broca neurons recognized as the theta rhythm generators eliminates theta oscillation in all connected cortical regions (68) and leads to spatial and working memory deficits (69–71).

Gamma oscillations ride on top of theta in the hippocampus and dynamically couple hippocampal networks to specific behavioral demands (39, 72, 73). The gamma oscillation is thought to be a readout of information transfer from CA3 to CA1, where CA3 is influencing the activity of CA1 for hippocampal memory retrieval to underlie memory encoding, consolidation, and episodic memory retrieval. Movement sequences, memory encoding and formation, sensory processing and planned trajectories underlying spatial navigation are thought to be temporally organized through timing mechanisms seen through gamma oscillations (74, 75).

The rate of populations of neuronal firing is also modulated in time in respect to theta frequency. For example, in the hippocampus, temporal modulation is manifested as burst firing with bursts occurring at theta frequency (4–12 Hz). The fidelity of burst firing within theta, termed theta modulation, is important for phenomena such as phase precession, phase preference and hippocampal replay, which are believed to allow encoding of space with higher resolution than is possible in the absence of modulation. Phase preference refers to the phenomenon that neuronal firing in the hippocampus is often locked in time with respect to ongoing hippocampal inputs in the theta frequency of the local field potential (LFP). Specific cells fire preferentially at specific phases in the ongoing theta oscillation. For example, directly after the peak of the theta oscillation, PV+ basket cells in CA2/3 area fire at the same phase as pyramidal cells in CA3, but later than basket cells in CA1 (76). CA1 pyramidal cells preferentially fire at the trough of theta (77, 78). Phase precession describes the observation that place cells will discharge whenever the rodent is crossing a place field and this firing occurs at an earlier phase in theta with progressive theta cycle (79, 80). This phase precession is believed to be an important component of information processing. Theta-phase precession could be an indication of item-context associations through spike timing-dependent plasticity (80, 81) given that synaptic inputs need to be precisely synchronized within 5 ms so that EPSPs from different locations will be able to induce postsynaptic firing (15). Umbach et al. showed that neurons in the hippocampus and entorhinal cortex not only fire for space, but also for time. Interestingly, time cells also exhibited theta-phase precession during memory encoding, and the activity of these cells correlates with the use of temporal location during the retrieval phase of the task (82). Neuronal firing coordination with the LFP, like phase-locking and phase precession, offers a key glimpse at the relative timing of inputs in the LFP with outputs of the information processing as the neuronal firing, but neural oscillations are also readouts of synchronized behavior of the network and, as such, are on their own important mesoscale mechanisms of cognition, memory, and behavior.

Coordination between oscillations seen in the LFP or EEG can be an indicator of communication between different brain regions (83, 84). Coherence is a measure of this synchronization with values that range between 0 and 1. The higher the coherence, the more synchronized the regions are (84). The coherence value differs between different brain regions depending on the task performed, for example, theta coherence between hippocampus and striatum during periods of decision is high (>0.8) which indicate learning (84, 85). Not only theta coherence changes during a task, but also gamma as well. Attention tasks in monkeys have shown that gamma coherence increases between the parietal and prefrontal areas (86). Furthermore, CA1 can become coherent with the entorhinal cortex or CA3 through fast or slow gamma characteristics of the entorhinal cortex or CA3, respectively (87). Elevation of hippocampal-entorhinal cortex synchrony was shown to be important for declarative memory formation in epileptic patients performing a memorization task (88). This led to the hypothesis that synchronized brain activity in the gamma range might be an important indicator of controlled flow and routing of information (83, 89), because the rules guiding synaptic plasticity dictate that inputs will be most effective whenever they coincide with peaks of oscillatory network excitability (83). Finally, neural coherence alterations were associated with different disorders like schizophrenia, attention-deficit hyperactivity disorder (ADHD), Alzheimer's disease (AD), and temporal lobe epilepsy (TLE) (90–95) (Table 2).

**Table 2:** Summary of cited clinical literature.

**Study**	**Subjects**	**Experiment**	**Main findings**
Spencer et al. (90)	Healthy and Schizophrenia Patients	Subjects responded whether an illusory square was present or absent in the trial along with EEG recording	•Abnormal phase-locking and abnormal phase coherence responses to the perception of an illusory visual stimuli in a Gestalt perception task that depends on neural synchrony •Abnormal neural circuit function may be an underlying cause of schizophrenia.
Herrmann and Demiralp (91)	Healthy and patients with ADHD, AD, epilepsy and schizophrenia	Gamma oscillations under various pathological conditions	•ADHD patients show an increase in gamma amplitudes •In Alzheimer's Disease (AD), there is a decrease in gamma response •In epileptic patients, there is an increase in gamma response which might be the readout of both cortical excitation and perceptual distortions •In schizophrenia patients, there is a decrease in gamma amplitude during negative symptoms, while there is an increase during positive symptoms such as hallucinations
Lega et al. (92)	Epilepsy patients	Recordings from hippocampal electrodes implanted in neurosurgical patients	•During successful episodic memory encoding there is an increase in the power of slow theta oscillations at 3 Hz •During successful memory encoding, there is a decrease in the fast theta hippocampal oscillation at 8 Hz
Barry and Clarke (93)	Children, adolescents, and adults with ADHD	Examine the resting-state EEG power and coherence, and event-related potentials (ERPs),	Different readouts that correlate with behavior and cognition: •Groups with high beta showed symptoms of increased delinquent behavior and reduced inattention, suicidal ideation, and physical problems. •Groups with elevated total power and theta and reduced alpha and beta showed fewer problems. •Groups with elevated slow wave activity and reduced alpha showed more impulsivity, inattention, and bad language. •Groups with reduced delta showed increased hyperactivity and ritualistic behaviors.
Wang et al. (95)	Healthy and AD patients	Recording resting eye-closed EEG signals followed by wavelet power spectrum and bicoherence of EEG analysis	•AD patients showed an increase in gamma and delta rhythms and a decrease in alpha power •The increase of the cross-frequency coupling strength between the beta/gamma and low-frequency bands in AD patients might be due to the disruption of GABAergic interneuron network showing an attenuated neuronal network

## Firing Dynamics Support Plasticity

Temporal, population, and rate coding facilitate plasticity shaped through experiences that enable the brain to adapt to new information. These mechanisms underlie the careful coordination of information between synapses and neurons in the brain that is necessary to promote synaptic plasticity and ensure efficient flow of information between different brain regions required for cognition. In 1949, Donald Hebb postulated that synapse strength can change based on previous activity, which led to what we now know as long-term potentiation (LTP) and long-term depression (LTD), fundamental to network communication. LTP strengthens synaptic transmission through high frequency stimulation of synapses. The first stage of LTP depends on the NMDA and AMPA glutamate receptors (96, 97). The second and third stages of LTP depend on protein synthesis to maintain changes in synaptic strength (96, 97). Maintenance of LTP is essential for place cell stability (98–101). Even though neural plasticity is not a determinant of place cell spatial specificity, rats with neural plasticity deficits had unstable place fields upon revisiting the same environment (102, 103). This shows that neural plasticity is playing a role in the organization of place cells and long-term maintenance of this representation.

The frequency of action potential timing matters; low frequency firing induces LTD, which decreases synaptic efficacy. LTD is also essential for memory formation as it counteracts the LTP to allow new memories to form. Recent evidence has shown that LTD may be involved in formation and maintenance of place fields (104), supporting previous experiments showing that a decrease in the expression of LTD impairs spatial memory retention and consolidation (105, 106). It is worth noting that plastic synapses can form positive feedback loops on the rate, temporal, and population coding mechanisms where this positive loop aids the mechanisms in refining and precisely timing the neuronal firing leading to a more efficient information processing. Another form of plasticity known as short-term plasticity (STP) takes place on a millisecond to minutes timescale and depends on presynaptic calcium accumulation and vesicle depletion (107). This form of plasticity is thought to play a role in information transfer across synaptic connections, activity-dependent synaptic efficacy modulation, promoting synchronization and working memory (107–109). Careful coordination of the firing of populations of neurons in time supports appropriate short and long-term forms of plasticity that are critical for information processing, learning and memory.

## Epilepsy and Memory

Cognitive impairments in people with epilepsy are extremely common and have a major negative influence on quality of life. Patients with focal epilepsy have shown to have a significant decrease in their quality of life compared to patients with generalized epilepsy and healthy controls, however, both focal and generalized epilepsy patients have a decreased self-esteem and increased anxiety compared to healthy controls (9). Identification of mechanisms of cognitive impairment is important as this will help to guide development of novel therapeutic strategies to improve outcomes. A major emphasis has been on studying the impact of seizures on cognition and exploring the epileptic encephalopathy hypothesis. Researchers have focused on the time of onset and frequency of seizures, however, there are few clear correlations between seizure characteristics and cognitive outcomes. Taken into consideration that cognitive functions are dependent on complex brain networks and both focal and generalized epilepsy groups share the same cognitive impairments (9), we can say that seizure location is less of a determinant of the cognitive impairments than an altered or dysfunctional network in the brain. This suggests that there must be other factors influencing cognitive outcomes in epilepsy. This assertion is supported by several lines of evidence. Studies assessing the effect of age of onset on cognitive impairment have shown that cognitive impairment already exists at pretreatment baseline in newly diagnosed children (3, 110–112). This led some researchers to question whether cognitive deficits could be present even before seizure onset (113) and this might suggest that the pre-existing impairments could be a result of the same dysregulation that underlies the seizures in the first place. Indeed, the need for special educational services prior to epilepsy onset is more common in children who were later diagnosed than those who weren't (114), suggesting that impairment in cognitive function may be present even before the first seizure. The best predictor of cognitive outcome up to 3 years after the diagnosis of epilepsy in infants is the initial cognitive profile, not any seizure or medication related factor. In adults, these impairments extend to deficits in visual motor tasks, mental flexibility, memory, reaction times, and attention (3, 115).

Others have studied the effects of the disorder duration on cognition, finding a negative correlation between the number of years of the disorder and brain volume (116, 117). This has been interpreted by some to mean that the length time since disease onset is related to the amount or significance of cognitive impairment. However, an alternate interpretation is that early onset of an epilepsy disorder is an indication of a more fundamentally dysfunctional network, leading to early development of recurrent seizures. Similarly, seizure frequency has been noted to be associated with a detrimental effect on cognition, with higher seizure frequency being correlated with lower performance on cognitive tasks and vice versa (9, 118). This was always interpreted to mean that seizures themselves were detrimental to cognition, rather than more frequent seizures being an indicator of a more abnormal neural network that underlies abnormal functioning during the interictal period manifesting as cognitive impairment. Hence, these impairments might not be due to age of onset or frequency of seizures, instead it could be attributed to the fact that children in this situation have a brain disease that presented earlier, and this difference in disease presentation may be an indicator of more severe network dysfunction, and more severe impairments. Further investigation of memory dysfunction in patients with epilepsy showed that people with epilepsy had significant deficits in both semantic and episodic autobiographical memory (9, 119, 120). This deficit was associated with young age at onset, more frequent seizures, and reduced working memory in early-onset epilepsy patients. In contrast, the same deficit was related to depression and lesion (3, 9, 120). The presence of both neurobiological and psychological factors suggests that information processing mechanisms might be altered (121).

Information processing through the mechanisms discussed in this review is shown to be altered in epilepsy and associated disorders. CA1 place cells are unstable in epileptic mice and undergo remapping a few weeks after pilocarpine-induced temporal lobe epilepsy (TLE). The number of place cells decreases, and the spatial tuning curve is less stable over time (122, 123). Prolonged recording over days from populations of neurons in CA1 and dentate gyrus has shown desynchronized interneuron firing between these two areas (124), which suggests that disruption of spatial coding is due to the loss of information processing control by interneurons. The desynchronized interneuron firing can affect the timing of the inputs being sent to the CA1. This was supported by the observation of theta rhythm temporal coordination loss in the dentate gyrus, where these neurons were firing at inconsistent phases of the CA1 theta rhythm (124). Spatial memory alteration was previously shown to be present even during the latent, seizure-free, period after either the pilocarpine-induced status epilepticus (SE) or early life seizures during the 1st weeks of life (39, 125, 126). These deficits were associated with a decrease in the power of theta oscillations (125). It is important to note that spontaneous seizures did not modify or affect any of the spatial deficits that were already present (125). Interestingly, Shuman et al. (124), found that place cell deterioration and place coding alteration occurred several weeks after pilocarpine induction, showing that the development of seizures is not solely responsible for place cell deterioration. Notably, place coding alteration, place cell deterioration, more dispersed place fields, and fewer place field responses were also seen after silencing either CA3, entorhinal cortex or both (124, 127, 128).

In addition, we and others have shown dysregulated population coding in epilepsy models. *In-vivo* single-unit recording showed that CA1 pyramidal cells are functionally connected to other pyramidal cells and fire in a coordinated fashion during spatial memory tasks; this connectivity is altered in TLE where neuronal reactivation and synchrony predicts the behavioral outcome in a TLE model (129). Population coding functional connectivity is also crucial within the hippocampus and between the hippocampus and PFC to underlie spatial working memory (SWM) (130, 131). During a SWM task, the hippocampal-PFC network shows a distributed dynamic code, seen through temporally regulated firing within and between brain regions, which is needed to combine separate processes together to execute a SWM task (131). The coordinated firing of cells in time is important for several components like attention, decision making and long-term memory, which can predict task performance. The temporal modulation of populations of neurons predicted SWM accuracy in a delayed non-match-to-sample task in control rats and rats with a cortical malformation that, in humans, is an important etiology in epilepsy. Animals with cortical malformations showed deficits in hippocampal firing modulation in addition to decreased functional connectivity between neurons (131).

Furthermore, population coding and neural dynamics are important for pattern separation and this process has been shown to be altered in hippocampal injury and epilepsy. The pattern separation depends on a network spanning different brain regions other than hippocampus, like the dorsal medial prefrontal cortex (dmPFC), however the hippocampus and the parahippocampal cortex serve as a hub for this network (132), thus it is expected that a hippocampal injury will alter the network communication causing pattern separation deficits (47, 133, 134). TLE patients and amnestic mild cognitive impairment (aMCI) patients have pattern separation deficits, and this could be due to hippocampal dysfunction involving DG and CA3 (46, 135). Another reason could be due to the failure of separating similar information during encoding by the hippocampus, hence memories will not be accurately encoded or retrieved. Studies investigating aMCI and TLE patients have shown that aMCI patients have an excess activation of the DG/CA3 area in fMRI compared to control groups and this excess activation is correlated with poor performance on pattern separation tasks. The same poor performance was seen in TLE patients performing the Mnemonic Similarity Task (MST). TLE patients demonstrated poor pattern separation performance compared to controls, however, it is important to note seizure and hippocampal sclerosis did not affect the performance of patients in this task (46, 133). Following studies showing that TLE patients have spatial mnemonic discrimination impairment and that TLE mice have DG-dependent object location memory deficits (50, 136), Madar et al. (52) tested pattern separation in TLE patients and mice with TLE, and then used mouse brain slices to record the spiking patterns of single granule cells (GC) in the dentate gyrus. TLE patients performing object recognition-based MST had a significant deficit in identifying similar but not identical objects suggesting that TLE might be impairing the DG-dependent mnemonic discrimination. Similar deficits were seen in mice with TLE as the mice had a decrease in object-location mnemonic discrimination compared to control mice (52). Slice electrophysiology in the same mice utlized inputs mimicking the same recorded inputs during behavior, and indicated that the output spike-trains of GCs had a higher average correlation compared to input correlation, which signifies a deficit in pattern separation in mice with TLE. Different input ranges demonstrated decreased pattern separation and convergence in DG at multiple timescale levels (52). This shows the importance of population dynamics underlying spatial deficits and signals the importance of assessing functional connectivity.

Imaging and histological experiments showed that structural and functional connectivity were altered in TLE patients as well (3, 137). Histological changes have been observed in the amygdala, entorhinal and parahippocampal cortices in TLE patients (3, 138–142). MRI images investigating hippocampal sclerosis associated with TLE, found that in addition to hippocampus, atrophy is present in the adjacent mesiotemporal, temporopolar structures, and thalamus (3, 143–146), and this atrophy increases over time (147–149). Experiments investigating tissue microstructure and structural covariance indicate that structural connectivity was impacted in TLE. Diffusion tensor MRI showed a disorganization in fiber arrangement in temporolimbic and adjacent regions (3, 150–153). Structural covariance such as cortical thickness or gray matter volume was altered between the mesiotemporal and neocortical regions and within the corticocortical networks (154–156). Resting state functional connectivity revealed a deficit in network connectivity in TLE patients compared to healthy controls. TLE patients had a decrease in ipsilateral mesiotemporal networks connectivity and ipsilateral and contralateral hippocampi connectivity (3, 153, 157–159). This decrease in connectivity extends beyond the temporal lobes into the posterior cingulate, inferior parietal, and medial prefrontal cortices disrupting the default mode network (DMN) (160–164). These changes and deficits suggest that structural connectivity is impacted in TLE patients and that TLE is also associated with functional connectivity deficits and reorganization.

Early stage TLE patients experience functional connectivity deficits mainly in the ipsilateral hemisphere (162, 165) in addition to disturbed interhemispheric connections (3, 158, 166). However, in patients with generalized epilepsy, there is an increase in the interhemispheric connectivity in addition to reduced functional connectivity (167–171). fMRI studies investigating the network connections in epileptic brains showed an increase in functional connectivity within the temporal lobe, alongside a decrease between temporal and other regions. Also, there is a decrease in the connection probability between neighboring brain regions, known as the clustering coefficient, within the DMN (161, 172). This decrease in clustering coefficient as well as increased path length, i.e., distance between one node and another, was revealed to be associated with cognitive decline in patients with cryptogenic epilepsy and only seen in patients with cognitive decline (172–174). The decreased cluster coefficient within the DMN could underlie the language impairment in patients with generalized epilepsy without focal brain damage. Gauffin et al. (175) conducted an experiment where patients with generalized epilepsy without focal damage performed a sentence-reading task while going through fMRI. Patients with generalized epilepsy took longer time to read both congruent (simple) and incongruent (complex) sentences compared to healthy controls with no reading time difference between congruent and incongruent sentences in the patients group which suggests that patients perceived both types as complex (175). BOLD fMRI indicated the activation of a left-lateralized frontotemporal network, anterior cingulate cortex and occipital cortex in both patients and controls upon reading both types of sentences (175). However, patients with generalized epilepsy had reduced DMN suppression compared to healthy controls (175). Further lack of suppression was seen in the left anterior temporal lobe and the posterior cingulate cortex, in addition to irregular activation of the right hippocampus proper and right parahippocampal gyrus (175). The reduced DMN activity suppression can be due to reduced functional segregation of the DMN in generalized epilepsy patients (170) where this can alter the balance between activated and deactivated neural networks hence disturbing the cognitive function (176, 177). Further evidence of network alteration in TLE patients was seen by Bernhardt et al. (178) upon analyzing hub nodes between controls and TLE patients. Hubs are also known as nodes that have multiple connections within a network with one central position and the connections formed by the hub nodes are essential for communication and network synchronization (179). Hub nodes in TLE patients were mainly located in the limbic and temporal association cortices instead of being evenly distributed between different lobes and this was thought to be due to connectivity disturbances between the temporolimbic and extratemporal neocortical structures (178) providing evidence that epileptic brains express decreased integration and enhanced segregation (172). It is also important to note that memory impairments are present in patients who don't show a lesion with MRI (180) which further supports the notion that cognitive impairments depend on the affected network rather than a structural lesion (9). These studies emphasize the necessity to move beyond the classical lesion model into a network approach which can provide several advantages by helping track or predict cognitive decline in epilepsy patients, improving diagnosis, and developing more accurate resection surgeries by targeting the areas where the hub nodes are mostly concentrated.

It is critical to note that experimental designs that induce an underlying disorder associated with epilepsy, but in which there are no overt seizures, and no other subclinical epileptiform activity was noted, show changes in information processing and behavioral deficits. Loss of function of sodium channels Nav1.1 associated with human epilepsy in CA1 can cause disruptions to place cells and spatial cognition without producing seizures (181). Nav1.1 knockdown in the medial septum causes alterations in temporal and rate coding in those neurons, and deficits in working memory that are correlated with the degree of LFP alteration in the hippocampus rather than seizure frequency (39, 182). Similar effects are seen in animals with a malformation of cortical development where no overt or subclinical seizures were noted. These animals have reduced fidelity of place cells, reduction in the magnitude of theta modulation, and disrupted population coding in addition to spatial and working memory deficits. The addition of induced seizures in this model did not make the behavioral deficits worse, indicating that the main contributor to the cognitive impairment was the underlying brain substrate and not seizures (183).

Notably, subclinical epileptiform activity or inter-ictal spikes (IIS) can disrupt cognitive function; however the number of spikes is not a reliable indicator of the associated cognitive impairment. Several studies have previously shown that patients with benign epilepsy with centro-temporal spikes experience IQ and school performance deficits (184, 185). These deficits were correlated with the frequency of IIS but not seizure frequency (184, 185). However, this may be related to timing of the IIS relative to ongoing cognitive processing, as the presence of the IIS may be an indicator that the brain is not in a state where it can be performing cognitive computations. Kleen et al. (186) investigated the effect of focal IIS on hippocampus in TLE. They showed that rats with unilateral intrahippocampal pilocarpine infusion developed hippocampal spikes that caused a response latency deficit in hippocampal-dependent operant behavior task, delayed-match-to-sample (186). However, the hippocampal spikes only altered the cognitive performance when they occur at the same time during memory retrieval; spikes occurring during memory encoding or maintenance did not affect the cognitive performance and overall IIS frequency during a trial was not predictive of accuracy during that trial (186). Similar results were seen in patients with refractory seizures performing Sternberg task, a delayed information task that depends on short-term memory processes, along with EEG recordings (187). Contralateral or bilateral to seizure focus hippocampal interictal epileptiform discharges (IED) during memory retrieval disrupted memory retrieval, and bilateral IED during memory maintenance was able to disrupt that process, however no effect was seen on memory encoding (187). These studies show that focal IIS and hippocampal IED are associated with disruptions in memory maintenance and retrieval only when they occur during the same time window as the memory processes. Taken together, this suggests that IIS/IED are indicators of disrupted network processing underlying cognition.

In addition to deficits in rate, temporal and population coding, plasticity deficits are also present in epilepsy, in accordance with the view that these neural coding mechanisms support plasticity. Kainic-acid induced status epilepticus (SE) model in rats shows a significant decrease in hippocampal LTP in addition to cell loss, and signs of hippocampal sclerosis (188). These rats also have deficits in the hippocampal-dependent novel object recognition spatial memory task that positively correlated with LTP magnitudes (188). These findings were also seen in the pilocarpine model where the mice showed a significant decrease in the hippocampal synaptopodin acting-binding protein in CA1 region which alters the ability of the neurons to express synaptic plasticity leading to a decrease in LTP induction in Schaffer collateral-CA1 synapses (189). STP and working memory are also altered in kainic acid-induced SE. Following kainic acid-induced SE, there was a decrease in STP, reduced LTP capacity, impaired spatial learning, and increased inhibition in the dentate gyrus (190). STP was altered in a model with recurrent hyperexcitability leading to seizures during development (191), as well as a model with aberrant GABA signaling during development leading to frequent interictal discharges. Animals with frequent IDs in the developing PFC showed a decrease in attention, and sociability alongside these changes in STP (192). The growing evidence on neural networks and epilepsy shows that these disrupted neural networks are likely responsible for the cognitive impairments seen with the disease and that the underlying etiology is the cause of both the disease and coding impairments seen in epilepsy animals and patients as well. The corollary is that recovering neural networks toward normal has potential for recovering cognitive impairments (Tables 3, 4).

**Table 3:** Overview of cited preclinical research.

**Study**	**Subjects**	**Experiment**	**Main findings**
Austin et al., Oostrom et al., and Berg et al. (110, 111, 114)	Children with new-onset seizures	Behavior ratings, behavior questionnaires and school records	Cognitive impairment exists at pretreatment baseline, special educational assistance required for newly diagnosed children, cognitive impairment present before the first seizure
Brun et al. (127)	Rats	MEC lesion	Place coding alteration, place cell deterioration, dispersed place fields, and less place field responses
Schlesiger et al. (193)	Rats	MEC lesion	Loss of theta phase precession in CA1
Hales et al. (194)	Rats	Bilateral MEC lesions	Place field and phase precession deficits, impaired spatial precision and spatial stability
Hernan et al. (131)	Rats	Malformation of cortical development	Hippocampal-PFC network shows less temporal modulation and less connectivity, underlying deficits in SWM
Karnam et al. (126)	Rats	ELS	Reduction in coherence, information content, center firing rate, and field size of place cells, instability of place fields, and spatial learning impairment
Hernan et al. (191, 192)	Rats	ELS/ early life IID	Increased STP in the PFC, decreased attention
Lynch et al. (190)	Rats	Kainic acid-induced SE	Decreased STP, reduced LTP capacity, impaired spatial learning, and increased inhibition in the dentate gyrus
Suárez et al. (188)	Rats	Kainic acid-induced SE	Significant decrease in hippocampal LTP, cell loss, signs of hippocampal sclerosis, and spatial memory task deficits
Ewell et al. (123)	Rats	Kainic acid-induced SE	Decreased number of active place cells, decreased spatial tuning curve stability
Liu et al. (122)	Rats	Pilocarpine SE/TLE	Decreased number of active place cells, decreased spatial tuning curve stability
Chauviere et al. (125)	Rats	Pilocarpine SE/TLE	Spatial memory alteration took place during seizure-free period and decreased theta oscillations power
Tyler et al. (129)	Rats	Pilocarpine SE/TLE	CA1 hippocampal pyramidal cells functional connectivity, coordinated firing, neuronal reactivation and synchrony predicts the behavioral outcome
Lenz et al. (189)	Mice	Pilocarpine SE/TLE	Significant decrease in the hippocampal synaptopodin acting-binding protein in CA1 region, decreased LTP induction in Schaffer collateral-CA1 synapses
Shuman et al. (124)	Mice	Pilocarpine SE	Desynchronized interneuron firing between CA1 and dentate gyrus, theta rhythm temporal coordination loss in the dentate gyrus, place cell deterioration and place coding alteration
Clawson et al. (121)	Rats	Pilocarpine SE	Storage and exchange of information, theta and slow oscillations disruption
Lenck-Santini and Holmes (195)	Rats	Hippocampal sclerosis/TLE	Phase precession and temporal organization disruption

**Table 4:** Main takeaways for the pre-clinical sections and associated clinical relevance.

**Coding**	**Physiology**		**Epilepsy**	
	**Preclinical**	**Clinical**	**Preclinical**	**Clinical**
Rate	• Place cells fire when an animal visits a specific place field • Time cells fire at specific times in a task called time fields and they can be time locked to an external stimulus • Grid cells provide activity-based maps of speed and direction in a certain environment and fire in different locations in an environment • Place and grid cells map are part of the greater hippocampal cognitive map • Inputs from the entorhinal cortex are important for hippocampal rate coding in the formation of the spatial memory and cognitive map • Selective disruption of the theta rhythm power correlated with spatial component of the non-verbal correlates of episodic-like memory task	• Time cells fire at specific times in a task called time fields and they can be time locked to an external stimulus • Inputs from the entorhinal cortex are important for hippocampal rate coding in the formation of the spatial memory and cognitive map	• Place cell misfiring • Loss of accurate spatial navigation • Lesioning the hippocampus results in loss of spatial memory • Lesioning the lateral entorhinal cortex impairs the hippocampal rate remapping upon changing the configuration of the environment • Time and grid cells deficits	• Time cells firing deficits • Disruption of entorhinal cortex inputs • Spatial memory deficits
Temporal	• The rate of populations of neuronal firing is also modulated in time • Temporal modulation is manifested as burst firing with bursts occurring at theta frequency in the hippocampus • Theta modulation is important for phase precession, phase preference and hippocampal replay • Phase precession is important for information processing. • Theta-phase precession could be an indication of item-context associations • Selective disruption of theta coordination across CA1 and the DG correlated with temporal component of the non-verbal correlates of episodic-like memory task	• The rate of populations of neuronal firing is also modulated in time • Neurons in the hippocampus and entorhinal cortex fire for space and time • Time cells exhibited theta-phase precession during memory encoding • Time cells activity correlates with the use of temporal location during retrieval phase of free recall task	• Loss of phase precession • Temporal modulation deficits • Item-context association deficits	• Loss of time modulation of neuronal firings • Theta-phase precession deficits • Temporal location alteration in free recall task
Population	• Neurons are functionally connected into a network • Population coding increases robustness of network function • Place cell populations will respond when the animal goes into the field • Dentate gyrus (DG) and its projection to CA3 underlie the pattern separation process • Working memory in the prefrontal cortex depends on population coding	• Pattern separation involves posterior occipitotemporal cortex (OTC) and the hippocampus • Dentate gyrus (DG) and its projection to CA3 underlie the pattern separation process • Working memory in the prefrontal cortex depends on population coding • BOLD signal on fMRI decreases during the delay phase of image-sequence matching task in humans • BOLD signal re-emerge during the image presentation phase of image-sequence matching task • Working memory information is maintained in the collective synaptic weights of populations of neurons in the PFC.	• Loss of functional connections • Decreased robustness of network function • Loss of place cells firing accuracy • DG aberrant CA3 influences • Working memory deficits	• Early stage TLE patients experience functional connectivity deficits in the ipsilateral hemisphere and interhemispheric connections • Patients with generalized epilepsy have an increase in the interhemispheric connectivity but reduced functional connectivity • Decreased cluster coefficient within the DMN underlies the language impairment in patients with generalized epilepsy without focal brain damage • Reduced DMN activity suppression can alter the balance between activated and deactivated neural networks and disturb cognitive function • Hub nodes in TLE patients were mainly located in the limbic and temporal association cortices instead of being evenly distributed between different lobes • Memory impairments are present in patients who don't show a lesion with MRI

## Therapeutic Strategies

The main issue with finding the appropriate treatment is whether neural network function can be recovered even in the context of a diseased brain. Here we will discuss potential therapeutic approaches that might influence the neural network function.

### Gene Therapy

Rett Syndrome (RTT) is a progressive neurodevelopmental disorder mainly affecting females in early childhood (196, 197). Development starts deteriorating at 6–18 months of age leading to neurological and neurobehavioral alterations and epilepsy (198). Loss of function mutations in the X-linked gene encoding the methyl-CpG-binding protein 2 (MeCP2) involved in transcriptional silencing and activation and RNA splicing modulation is thought to contribute to the pathophysiology of RTT (199, 200).

RTT is associated with significant behavioral abnormalities: motor discoordination and social interaction deficits as well as deficits in cognitive abilities like learning and memory (201–203). MeCP2 knockout mice show reduced neuronal activity in cortical and hippocampal areas (204) as well as deficits in LTP expression in the hippocampus (197). Epilepsy has been reported in 60–80% of RTT patients (205–207). Although children with RTT often have seizures, it is widely accepted that the main driver of the cognitive impairments is a function of the genetic cause. Deficits in LTP, reduction in neuronal activity and seizures indicate that behavioral and cognitive deficits extend to a network problem that involves several mechanisms underlying neuronal activity and plasticity.

Hippocampal place cells are impaired in RTT mice (203). Normally, place fields become refined as the animal-environment experience increases and are stabilized during memory consolidation in sleep. This process involves synchronous re-activation within high-frequency short time-scale windows, known as sharp-wave ripples (208), which is associated with synaptic plasticity transforming short-term memories into long-term ones (209). This process is disrupted in RTT mice as these mice show deficits in experience-dependent refinement of spatial information in addition to increased place cell baseline firing synchrony during sleep (203). Neural oscillations are also impaired in RTT. Organoids developed from stem cells of RTT patients, demonstrated individual neuron firing at a rapid and persistent rate, diminished or reduced gamma oscillation in addition to epileptiform-appearing spikes and high-frequency oscillations (210, 211). Rett mice show desynchronized and reduced theta oscillations during exploratory behavior (210, 212), underscoring impaired temporal coding underlying cognitive and behavioral deficits in RTT mice.

MeCP2 gene therapy has been shown to improve the survival and improve some behavioral deficits seen in RTT (196). Treated mice showed normalized gene expression in addition to better mobility and more exploratory behavior in the open field (213, 214), which could involve normalized place cell activity. This improvement was accompanied by a normalization of neuronal nuclear volume in MeCP2 transduced cells in the dentate gyrus (215). MeCP2 is a master transcriptional regulator of activity-dependent gene expression; recovering it may restore the brain's ability to respond plastically, thereby allowing the network to be in a state where it is ready to receive new information.

Rett syndrome is a very specific disorder whose pathophysiology seems to be directly related to MeCP2. Other causes of epilepsy are less straightforward and may require other gene therapy strategies. One such strategy is targeting the hyperexcitable granule cells in the dentate gyrus in TLE (216). Reducing granule cell hyperactivity *via* inhibitory chemogenetic receptors, DREADDs (CamKIIα-hM4Di), was able to normalize performance in the spatial object recognition task, reduce seizures and restore the dentate gyrus information coding process (216). Over-expression of the voltage-gated potassium channel Kv1.1 *via* lentiviral vector or AAV significantly reduced the seizure frequency in rats with focal neocortical epilepsy (FNE) or TLE, respectively (217). Evidence from behavioral and cognitive studies in epilepsy emphasize the need for a new gene therapy strategies. Cognitive and behavioral deficits vary among epilepsy patients, even patients with the same type of epilepsy as this could be due to different genomic factors (3, 218). Different genetic variants are associated with various comorbidities. For example, executive dysfunction was associated with catechol-O-methyltransferase (COMT), methylenetetrahydrofolate reductase (MTHFR) and BDNF in TLE and pediatric epilepsy (219, 220), memory impairment was associated with apolipoprotein E (APOE) and BDNF in TLE (221, 222), impaired working memory was associated with COMT and MTHFR in pediatric epilepsy (220), decreased information processing was associated with RE1- silencing transcription factor (REST) (219), and anxiety and depression were associated with BDNF and COMT (223). Further investigation of epigenomic, transcriptomic, and proteomic changes in epilepsy along with understanding the functional recovery mechanisms seen with gene therapy in RTT, TLE, and FNE at the level of rate, population and temporal coding will allow us to explore the possibility of treating diseased brains.

### Environmental Enrichment

The efficacy of simple environmental enrichment (EE) strategies on improving cognition was first noted by Donald Hebb in 1947. He found that the rats he took home with him performed better on behavioral tasks than rats housed in the lab (224). This spurred Hebb's hypothesis that frequent pairing of neuronal firing leads to more efficient excitation in the future; that exposure to a more enriched environment during development critical periods might be influencing the behavior in adulthood. Hebb's observations were the first to connect environmental influences to plasticity. Today, EE paradigms involve exposure to different housing conditions that enable sensory, motor, and cognitive stimulation (225, 226).

EE has been studied in different neurological diseases like Parkinson's and Alzheimer's diseases. EE has been shown to slow cognitive decline in Alzheimer's disease (227). To investigate the effects of EE on epilepsy, it was shown that EE can reduce cognitive deficits, increase neural plasticity, improve motor coordination, and reduce the frequency of seizures (228). We will focus on the effects of EE on information processing mechanisms.

There is a strong link between EE, plasticity, and the mechanisms underlying plasticity. Housing young rats in an enriched environment for 30 days was shown to increase synaptophysin and post-synaptic density (PSD) in the cortex, hippocampus, thalamus, and hypothalamus (226). This suggests that the enriched environment was able to stimulate the formation of new functional synapses in these brain regions. Hippocampal gamma power increases during theta states in rats housed in enriched environments (229). This occurs alongside an increase in interhemispheric coherence of gamma oscillations after EE (229). Mice housed in EE conditions also had an increase in CA1 gamma oscillations (230).

EE also affects rate and population coding. Prolonged exposure to an enriched environment was able to increase the selectivity of CA1 place cells to a particular area in the arena in a way where fewer place cells are activated after brief exposure to a novel environment, along with an increase in global remapping efficiency and this was further supported by the increased expression of the activation protein Arc in CA1 and dentate gyrus (231). This might suggest that the exposure to an enriched environment might be changing how place cells process information by recruiting more or new populations of neurons leading to a more efficient population coding mechanism.

Due to the various effects of EE on these important mechanisms, interest has been growing in investigating the effects of EE on epilepsy. Exposing rats with absence epilepsy to an enriched environment resulted in fewer seizures in adulthood, reduced seizure frequency, and reduced anxiety levels in adulthood (232). The beneficial effects of EE were also seen in TLE rats in the lithium/pilocarpine model. EE was able to alleviate depression and hyperactivity in addition to restoring theta LFP power in the CA1 region (233). The positive effects of EE were further seen in rats with malformation of cortical development (MCD). Rats with MCD had a disruption in their fine spike timing and place-modulated rate coding in CA1 region, which was improved upon with EE exposure (234).

These studies show that EE have a positive impact on rate and population coding. This is important as these processes are disrupted in epilepsy where these are essential for information processing and plasticity. This opens the door for future investigations on how EE can possibly modulate the brain network in ways that make it less susceptible to insults and improves outcome in patients with epilepsy.

### Brain Stimulation

Brain stimulation is another therapeutic option for improving cognitive deficits associated with a variety of neurological diseases. Brain stimulation can either activate or inhibit the brain activity in a specific region which gives the ability to modulate cognitive functions. Various types of brain stimulation exist, deep brain stimulation (DBS) is an invasive technique that involves direct implantation of electrodes in the brain while transcranial magnetic stimulation (TMS) is a non-invasive technique that uses magnetic fields applied to the head (235). Brain stimulation techniques have been mainly studied in Alzheimer's disease (AD) and Parkinson Disease (PD).

DBS was tested in AD for the first time in 1984, and while this study did not show any memory or cognitive improvements, it was able to partially stop the left frontal lobe deterioration (236). In 2010, DBS went into phase I trial to investigate its effect on AD patients, and it was shown that after DBS of the fornix/hypothalamus, the patients had improved memory, reduced cognitive decline, enhanced mental state and social performance in addition to increased hippocampal volume (237–239). Further experiments exploring DBS and AD took place after this trial, and the experiments showed the positive effects of DBS on stabilizing cognitive performance (240), influencing cognitive function and disease progression depending on the disease stage and brain region being stimulated (241). For example, nucleus basalis of Meynert (NBM) DBS had a positive effect on sensory gating of auditory information into memory (242). Repetitive TMS (rTMS) was also applied for AD patients. rTMS delivers trains of pulses at the same intensity over a period of time. It mainly uses high frequency (≥5 Hz) for cortical excitability, low-frequency ( ≤ 1 Hz) for cortical inhibition or theta-burst stimulation (TBS) (243). Several trials have shown that rTMS enhanced cognitive function in AD patients when applied to the bilateral dorsolateral prefrontal cortices (DLPFCs) (244–247). Animal studies also investigated the effect of DBS on AD. Acute fornix DBS was able to improve learning and long-term memory in the triple transgenic AD mouse (3 × Tg) model (248). Also, bilateral intermittent NBM DBS enhanced and maintained spatial memory tasks in AD rats (249). Similar results were seen with single rostral intralaminar thalamic (ILN) DBS, in addition to preservation of dendritic spine density in the mPFC and hippocampus and enhanced expression of PSD-95 (250).

In PD, bilateral subthalamic nucleus (STN) and internal globus pallidus (GPi) DBS was able to significantly reduce dyskinesia and improve motor symptoms with long-term benefit (251–253). Additional studies have shown overall improvement in quality of life and continued efficacy in patients that lasted more than 10 years (254, 255). Although most studies agree on the positive effects of DBS on motor function and quality of life, there is contradictory evidence on the positive effects of DBS on cognition and attention in PD patients. Some studies have found that PD patients continued to experience PD-associated declines in executive function, visuospatial reasoning and memory, and verbal memory after DBS (256–258). However, other studies have shown that DBS groups performed better than control groups in memory functions and visuospatial tasks (259, 260). The contradictory results seen with DBS on cognition in PD patients could be due to the stimulated brain regions and using on paradigm for all patients. STN and GPi are the most studied regions in PD due to their importance in dyskinesia and motor coordination, however these regions are not directly involved in memory *per se*.

DBS is used for epilepsy patients to control and manage refractory seizures; however, DBS may also be beneficial for the cognitive deficits seen in the patients. Ezzyat et al. developed a subject-based approach to investigate the effect of DBS on memory facilitation if performed in a timely manner. Taking into account the disrupted memory network in epilepsy patients, interfering at the right time can reverse the dysfunctional activity of memory encoding. The team was able to differentiate low from high encoding states which indicate neural activity and either stimulating a single medial temporal lobe (MTL) structure like hippocampus or structure involved in memory encoding like prefrontal cortex in the learning session (261). Studies stimulating a single MTL region had contradictory conclusions, indicating both memory facilitation (262, 263) and memory disruption (264, 265). Interestingly, the stimulation was able to increase the encoding-state and memory recall when performed during low-encoding states (261) and this suggests that the accurate stimulation of a single MTL structure or a region involved in memory encoding can reverse the deficits if done at a specific time of memory process. Pilocarpine rats showed a decrease in hippocampal theta power and percentage of time oscillating in theta (266), however, continuous stimulation through Barnes maze task or pre-task stimulation of the medial septum at 7.7 Hz was able to prevent theta oscillations reductions, improve spatial navigation and search strategy during the task. This cognitive improvement was accompanied by significant increase in seizure threshold in these rats. This shows that theta stimulation of the septum has potential to rescue cognitive impairments and increase seizure threshold, further supporting a mechanistic link upstream of both of these symptoms of epilepsy (266, 267). The same stimulation paradigm was used with rats after a traumatic brain injury (TBI) and it was shown that these rats had improved spatial learning and object exploration in addition to increased hippocampal theta oscillations (268). Taken together, these data show that neuronal stimulation approaches may be effective in restoring normal network function and improving cognition broadly.

### Interneuron Implantation

Interneuron implantation is another possible treatment that can potentially recover the network function given the importance of interneurons in balancing the inhibition-excitation, controlling gamma and theta oscillations, and sharp wave ripples in the hippocampus. Interneuron precursor implantation into the prefrontal cortex of Pten mutant mice, an autism mouse model, was able to reverse the social behavior deficits seen in these mice; however, the implantation did not normalize baseline and social interaction-evoked EEG signals, but did modify inhibitory signaling in the PFC, underscoring a complex relationship between etiology and circuit restoration underlying behavioral improvement in disease (269). Interneuron implantation has been shown to be beneficial in epilepsy as well. Implantation in TLE, absence epilepsy, and generalized epilepsy models in rodents was able to increase seizure threshold, reduce seizure frequency and duration, reduce network excitability, and improve behavioral deficits (270–273). Implanting interneurons derived from human induced pluripotent stem cell (hiPSC) into the hippocampus of TLE rat model was able to reduce spontaneous seizures frequency after status epilepticus (274–276) which shows translational significance from rodents to humans. In addition to reducing seizures frequency, there was a decrease in the aberrant mossy fiber sprouting, and improved cognition and mood. The implanted rats showed an improvement in hippocampal dependent tasks like object recognition and improvement in pattern separation and novel object recognition (276), which suggests that the implantation might be recovering the communication between different regions or reactivating the DG/CA3 connections required for pattern separation. Integration of interneurons into the CA3 network may be how the new interneurons are affecting the network. region of the hippocampus of epileptic mice was able to improve the working memory in Y-maze test and spatial memory in water maze, however both tasks depend on the PFC (270, 274, 277). This raises the question of how locally implanted interneurons can enhance tasks that are dependent on different brain regions as well. Given the crucial role for interneurons in the timing of the action potential firing, these local connections are likely refining the signal from hippocampus to the PFC. Interestingly, MGE implantation was able to increase memory precision in mice with traumatic brain injury (TBI) as well. Implanted mice performed better in object location task and contextual fear memory where both tasks depend on hippocampus and hippocampal interneurons, respectively (278). Based on these data, GABAergic interneurons transplants may be a promising therapeutic approach for different diseases, however, further investigations are needed to determine the right time and location of implantation for the different investigated diseases.

## Conclusion

In this review, we addressed the role of neuronal dynamics in supporting proper cognition, learning and memory, and discussed how these dynamics are altered in epilepsy. The data suggest that cognitive impairments seen in patients with epilepsy and preclinical models of epilepsy are likely due to plasticity changes, alterations to neuronal coding regimes, desynchronization, and functional connectivity disruptions from the effect of underlying etiology, rather than seizures themselves. Although we accept that the seizures could also have some negative impact on network behaviors, we strongly argue that the seizure effect is very small when compared to the etiology effect. We therefore suggest that these deficits should be approached from a systems neuroscience perspective, while being informed by mechanisms needed for normal cognitive function and development in a dynamic experience-dependent and plastic network. Importantly, this calls us to move beyond seizures into network science that is guiding possible treatments and defining new pathophysiology. This might help advance the epilepsy research forward and open the door potentially to answer unsolved questions in the field.

## Author Contributions

MK, AH, and RS: draft manuscript writing and editing. All authors reviewed the final draft manuscript. All authors contributed to the article and approved the submitted version.

## Funding

AH and MK were funded by an NIH NINDS K22NS104230. RS was funded by an NIH NINDS R21NS117112.

## Conflict of Interest

The authors declare that the research was conducted in the absence of any commercial or financial relationships that could be construed as a potential conflict of interest.

## Publisher's Note

All claims expressed in this article are solely those of the authors and do not necessarily represent those of their affiliated organizations, or those of the publisher, the editors and the reviewers. Any product that may be evaluated in this article, or claim that may be made by its manufacturer, is not guaranteed or endorsed by the publisher.

## References

[1] Fisher RS Acevedo C Arzimanoglou A Bogacz A Cross JH Elger CE. ILAE official report: a practical clinical definition of epilepsy. Epilepsia. (2014) 55:475–82. 10.1111/epi.12550 24730690

[2] Cano-López I Hampel KG Garcés M Villanueva V González-Bono E. Quality of life in drug-resistant epilepsy: relationships with negative affectivity, memory, somatic symptoms and social support. J Psychosom Res. (2018) 114:31–7. 10.1016/j.jpsychores.2018.09.001 30314576

[3] Hermann BP Struck AF Busch RM Reyes A Kaestner E McDonald CR. Neurobehavioural comorbidities of epilepsy: towards a network-based precision taxonomy. Nat Rev Neurol. (2021) 17:731–46. 10.1038/s41582-021-00555-z 34552218 PMC8900353

[4] Gauffin H Landtblom A-M Räty L. Self-esteem and sense of coherence in young people with uncomplicated epilepsy: a 5-year follow-up. Epilepsy Behav. (2010) 17:520–4. 10.1016/j.yebeh.2010.01.167 20227922

[5] Winter DG. Personality: Analysis and Interpretation of Lives. New York, NY: McGraw-Hill (1996). p. 678.

[6] Antonovsky A. Unraveling the Mystery of Health: How People Manage Stress and Stay Well. San Francisco, CA: Jossey-Bass (1987). p. 218.

[7] Gauffin H Flensner G Landtblom A-M. Living with epilepsy accompanied by cognitive difficulties: young adults' experiences. Epilepsy Behav. (2011) 22:750–8. 10.1016/j.yebeh.2011.09.007 22019020

[8] Fisher RS. The new classification of seizures by the international league against epilepsy 2017. Curr Neurol Neurosci Rep. (2017) 17:48. 10.1007/s11910-017-0758-6 28425015

[9] Gauffin H Landtblom A.-M. Vigren P Frick A Engström M McAllister A. Similar profile and magnitude of cognitive impairments in focal and generalized epilepsy: a pilot study. Front Neurol. (2022) 12:746381. 10.3389/fneur.2021.746381 35095714 PMC8790571

[10] Bell B Lin JJ Seidenberg M Hermann B. The neurobiology of cognitive disorders in temporal lobe epilepsy. Nat Rev Neurol. (2011) 7:154–64. 10.1038/nrneurol.2011.3 21304484 PMC3856217

[11] Bartha-Doering L Trinka E. The interictal language profile in adult epilepsy. Epilepsia. (2014) 55:1512–25. 10.1111/epi.12743 25110150

[12] Loughman A Bowden SC D'Souza WJ. A comprehensive assessment of cognitive function in the common genetic generalized epilepsy syndromes. Eur J. Neurol. (2017) 24:453–60. 10.1111/ene.13232 28026919

[13] Simani L Roozbeh M Rostami M Pakdaman H Ramezani M Asadollahi M. Attention and inhibitory control deficits in patients with genetic generalized epilepsy and psychogenic nonepileptic seizure. Epilepsy Behav. (2020) 102:106672. 10.1016/j.yebeh.2019.106672 31739099

[14] Gerstner W Kreiter AK Markram H Herz AVM. Neural codes: firing rates and beyond. Proc Nat Acad Sci USA. (1997) 94:12740–1. 10.1073/pnas.94.24.12740 9398065 PMC34168

[15] Lenck-Santini P-P Scott RC. Mechanisms responsible for cognitive impairment in epilepsy. Cold Spring Harb Perspect Med. (2015) 5:a022772. 10.1101/cshperspect.a022772 26337111 PMC4588128

[16] Zuo Y Safaai H Notaro G Mazzoni A Panzeri S Diamond ME. Complementary contributions of spike timing and spike rate to perceptual decisions in rat S1 and S2 cortex. Curr Biol. (2015) 25:357–63. 10.1016/j.cub.2014.11.065 25619766

[17] Dragoi G Harris KD Buzsáki G. Place representation within hippocampal networks is modified by long-term potentiation. Neuron. (2003) 39:843–53. 10.1016/S0896-6273(03)00465-3 12948450

[18] Lu L Leutgeb JK Tsao A Henriksen EJ Leutgeb S Barnes CA. Impaired hippocampal rate coding after lesions of the lateral entorhinal cortex. Nat Neurosci. (2013) 16:1085–93. 10.1038/nn.3462 23852116

[19] Tolman EC. Cognitive maps in rats and men. Psychol Rev. (1948) 55:189–208. 10.1037/h0061626 18870876

[20] O'Keefe J Nadel L. The Hippocampus as a Cognitive Map. Oxford; New York, NY: Clarendon Press; Oxford University Press (1978).

[21] Davidson TJ Kloosterman F Wilson MA. Hippocampal replay of extended experience. Neuron. (2009) 63:497–507. 10.1016/j.neuron.2009.07.027 19709631 PMC4364032

[22] Clark RE Broadbent NJ Squire LR. Impaired remote spatial memory after hippocampal lesions despite extensive training beginning early in life. Hippocampus. (2005) 15:340–6. 10.1002/hipo.20076 15744736 PMC2754396

[23] Pastalkova E Itskov V Amarasingham A Buzsáki G. Internally generated cell assembly sequences in the rat hippocampus. Science. (2008) 321:1322–7. 10.1126/science.1159775 18772431 PMC2570043

[24] Hafting T Fyhn M Molden S Moser M-B Moser EI. Microstructure of a spatial map in the entorhinal cortex. Nature. (2005) 436:801–6. 10.1038/nature03721 15965463

[25] O'Keefe J Burgess N. Dual phase and rate coding in hippocampal place cells: theoretical significance and relationship to entorhinal grid cells. Hippocampus. (2005) 15:853–66. 10.1002/hipo.20115 16145693 PMC2677681

[26] Muller R Kubie J. The effects of changes in the environment on the spatial firing of hippocampal complex-spike cells. J Neurosci. (1987) 7:1951–68. 10.1523/JNEUROSCI.07-07-01951.1987 3612226 PMC6568940

[27] Ruth RE Collier TJ Routtenberg A. Topographical relationship between the entorhinal cortex and the septotemporal axis of the dentate gyrus in rats: II. Cells projecting from lateral entorhinal subdivision. J Comparat. Neurol. (1988) 270:506–16. 10.1002/cne.902700404 2836479

[28] Pouget A Dayan P Zemel R. Information processing with population codes. Nat Rev Neurosci. (2000) 1:125–32. 10.1038/35039062 11252775

[29] Usrey WM Reid RC. Synchronous activity in the visual system. Annu Rev Physiol. (1999) 61:435–56. 10.1146/annurev.physiol.61.1.435 10099696

[30] Tolhurst DJ Movshon JA Dean AF. The statistical reliability of signals in single neurons in cat and monkey visual cortex. Vision Res. (1983) 23:775–85. 10.1016/0042-6989(83)90200-6 6623937

[31] Salinas E Abbott LF. Vector reconstruction from firing rates. J Comput Neurosci. (1994) 1:89–107. 10.1007/BF00962720 8792227

[32] Atallah BV Bruns W Carandini M Scanziani M. Parvalbumin-expressing interneurons linearly transform cortical responses to visual stimuli. Neuron. (2012) 73:159–70. 10.1016/j.neuron.2011.12.013 22243754 PMC3743079

[33] Adesnik H. Synaptic mechanisms of feature coding in the visual cortex of awake mice. Neuron. (2017) 95:1147–59.e4. 10.1016/j.neuron.2017.08.014 28858618 PMC5580349

[34] Adesnik H. Layer-specific excitation/inhibition balances during neuronal synchronization in the visual cortex. J Physiol. (2018) 596:1639–57. 10.1113/JP274986 29313982 PMC5924838

[35] Aertsen A Braitenberg V. Brain Theory: Biological Basis Computational Principles. Amsterdam; New York, NY: Elsevier. (1996). Available online at: http://site.ebrary.com/id/10254720 (accessed February 14, 2022).

[36] Anderson JS Carandini M Ferster D. Orientation tuning of input conductance, excitation, and inhibition in cat primary visual cortex. J Neurophysiol. (2000) 84:909–26. 10.1152/jn.2000.84.2.909 10938316

[37] Li Y Liu B Chou X Zhang LI Tao HW. Synaptic basis for differential orientation selectivity between complex and simple cells in mouse visual cortex. J Neurosci. (2015) 35:11081–93. 10.1523/JNEUROSCI.5246-14.2015 26245969 PMC4524977

[38] Li Y Ma W Pan C Zhang LI Tao HW. Broadening of cortical inhibition mediates developmental sharpening of orientation selectivity. J Neurosci. (2012) 32:3981–91. 10.1523/JNEUROSCI.5514-11.2012 22442065 PMC3372461

[39] Trevelyan AJ Bruns W Mann EO Crepel V Scanziani M. The information content of physiological and epileptic brain activity. J Physiol. (2013) 591:799–805. 10.1113/jphysiol.2012.240358 23027823 PMC3591698

[40] Fu Y Tucciarone JM Espinosa JS Sheng N Darcy DP Nicoll RA. A cortical circuit for gain control by behavioral state. Cell. (2014) 156:1139–52. 10.1016/j.cell.2014.01.050 24630718 PMC4041382

[41] Pakan JM Lowe SC Dylda E Keemink SW Currie SP Coutts CA. Behavioral-state modulation of inhibition is context-dependent and cell type specific in mouse visual cortex. Elife. (2016) 5:e14985. 10.7554/eLife.14985 27552056 PMC5030095

[42] Millman DJ Ocker GK Caldejon S Kato I Larkin JD Lee EK. VIP interneurons in mouse primary visual cortex selectively enhance responses to weak but specific stimuli. eLife. (2020) 9:e55130. 10.7554/eLife.55130 33108272 PMC7591255

[43] Rubin DB Van Hooser SD Miller KD. The stabilized supralinear network: a unifying circuit motif underlying multi-input integration in sensory cortex. Neuron. (2015) 85:402–17. 10.1016/j.neuron.2014.12.026 25611511 PMC4344127

[44] França TFA Monserrat JM. Hippocampal place cells are topographically organized, but physical space has nothing to do with it. Brain Struct Funct. (2019) 224:3019–29. 10.1007/s00429-019-01968-9 31654118

[45] Lohnas LJ Duncan K Doyle WK Thesen T Devinsky O Davachi L. Time-resolved neural reinstatement and pattern separation during memory decisions in human hippocampus. Proc Nat Acad Sci USA. (2018) 115:E7418–27. 10.1073/pnas.1717088115 30006465 PMC6077719

[46] Lalani SJ Reyes A Kaestner E Stark SM Stark CEL Lee D. Impaired behavioral pattern separation in refractory temporal lobe epilepsy and mild cognitive impairment. J Int Neuropsychol Soc. (2021) 2021:1–13. 10.1017/S1355617721000734 34078506 PMC8965747

[47] Leutgeb JK Leutgeb S Moser M-B Moser EI. Pattern separation in the dentate gyrus and CA3 of the hippocampus. Science. (2007) 315:961–6. 10.1126/science.1135801 17303747

[48] Bakker A Kirwan CB Miller M Stark CEL. Pattern separation in the human hippocampal CA3 and dentate gyrus. Science. (2008) 319:1640–2. 10.1126/science.1152882 18356518 PMC2829853

[49] Wesnes KA Annas P Basun H Edgar C Blennow K. Performance on a pattern separation task by Alzheimer's patients shows possible links between disrupted dentate gyrus activity and apolipoprotein E 4 status and cerebrospinal fluid amyloid-β42 levels. Alzheimer's Res Ther. (2014) 6:20. 10.1186/alzrt250 24735568 PMC4054957

[50] Reyes A Holden HM Chang Y-HA Uttarwar VS Sheppard DP DeFord NE. Impaired spatial pattern separation performance in temporal lobe epilepsy is associated with visuospatial memory deficits and hippocampal volume loss. Neuropsychologia. (2018) 111:209–15. 10.1016/j.neuropsychologia.2018.02.009 29428769 PMC5873595

[51] Madar AD Ewell LA Jones MV. Pattern separation of spiketrains in hippocampal neurons. Sci Rep. (2019) 9:5282. 10.1038/s41598-019-41503-8 30918288 PMC6437159

[52] Madar AD Pfammatter JA Bordenave J Plumley EI Ravi S Cowie M. Deficits in behavioral and neuronal pattern separation in temporal lobe epilepsy. bioRxiv:2020.02.13.948364. (2020). 10.1101/2020.02.13.948364 PMC8612476 34620720

[53] Baddeley AD Logie RH. Working memory: the multiple-component model. In: A Miyake, P Shah, editors, *Models of Working Memory*. 1st ed. Cambridge: Cambridge University Press (1999). p. 28–61. 10.1017/CBO9781139174909.005

[54] Sreenivasan KK Curtis CE D'Esposito M. Revisiting the role of persistent neural activity during working memory. Trends Cogn Sci. (2014) 18:82–9. 10.1016/j.tics.2013.12.001 24439529 PMC3964018

[55] Sarma A Masse NY Wang X-J Freedman DJ. Task-specific versus generalized mnemonic representations in parietal and prefrontal cortices. Nat Neurosci. (2016) 19:143–9. 10.1038/nn.4168 26595652 PMC4880358

[56] Masse NY Hodnefield JM Freedman DJ. Mnemonic encoding and cortical organization in parietal and prefrontal cortices. J Neurosci. (2017) 37:6098–112. 10.1523/JNEUROSCI.3903-16.2017 28539423 PMC5481944

[57] Emrich SM Riggall AC LaRocque JJ Postle BR. Distributed patterns of activity in sensory cortex reflect the precision of multiple items maintained in visual short-term memory. J Neurosci. (2013) 33:6516–23. 10.1523/JNEUROSCI.5732-12.2013 23575849 PMC3664518

[58] Wasmuht DF Spaak E Buschman TJ Miller EK Stokes MG. Intrinsic neuronal dynamics predict distinct functional roles during working memory. Nat Commun. (2018) 9:3499. 10.1038/s41467-018-05961-4 30158572 PMC6115413

[59] Pinto L Dan Y. Cell-type-specific activity in prefrontal cortex during goal-directed behavior. Neuron. (2015) 87:437–50. 10.1016/j.neuron.2015.06.021 26143660 PMC4506259

[60] Murray JD Bernacchia A Roy NA Constantinidis C Romo R Wang X-J. Stable population coding for working memory coexists with heterogeneous neural dynamics in prefrontal cortex. Proc Nat Acad Sci USA. (2017) 114:394–9. 10.1073/pnas.1619449114 28028221 PMC5240715

[61] Buzsáki G Draguhn A. Neuronal oscillations in cortical networks. Science. (2004) 304:1926–9. 10.1126/science.1099745 15218136

[62] Wang X-J. Neurophysiological and computational principles of cortical rhythms in cognition. Physiol Rev. (2010) 90:1195–268. 10.1152/physrev.00035.2008 20664082 PMC2923921

[63] Buzsáki G. Theta oscillations in the hippocampus. Neuron. (2002) 33:325–40. 10.1016/S0896-6273(02)00586-X 11832222

[64] Buzsáki G. Theta rhythm of navigation: link between path integration and landmark navigation, episodic and semantic memory. Hippocampus. (2005) 15:827–40. 10.1002/hipo.20113 16149082

[65] Mitchell SJ Ranck JB. Generation of theta rhythm in medial entorhinal cortex of freely moving rats. Brain Res. (1980) 189:49–66. 10.1016/0006-8993(80)90006-2 7363097

[66] Leung L-WS Borst JGG. Electrical activity of the cingulate cortex. I. Generating mechanisms and relations to behavior. Brain Res. (1987) 407:68–80. 10.1016/0006-8993(87)91220-0 3580857

[67] Paré D Collins DR. Neuronal correlates of fear in the lateral amygdala: multiple extracellular recordings in conscious cats. J Neurosci. (2000) 20:2701–10. 10.1523/JNEUROSCI.20-07-02701.2000 10729351 PMC6772231

[68] Petsche H Stumpf C Gogolak G. The significance of the rabbit's septum as a relay station between the midbrain and the hippocampus I. The control of hippocampus arousal activity by the septum cells. Electroencephal Clin Neurophysiol. (1962) 14:202–11. 10.1016/0013-4694(62)90030-5 14038334

[69] Smith HR Pang KCH. Orexin-saporin lesions of the medial septum impair spatial memory. Neuroscience. (2005) 132:261–71. 10.1016/j.neuroscience.2004.12.037 15802181

[70] Dwyer TA Servatius RJ Pang KCH. Noncholinergic lesions of the medial septum impair sequential learning of different spatial locations. J Neurosci. (2007) 27:299–303. 10.1523/JNEUROSCI.4189-06.2007 17215389 PMC3063940

[71] Cañas A Juncadella M Lau R Gabarrós A Hernández M. Working memory deficits after lesions involving the supplementary motor area. Front Psychol. (2018) 9:765. 10.3389/fpsyg.2018.00765 29875717 PMC5974158

[72] Montgomery SM Buzsaki G. Gamma oscillations dynamically couple hippocampal CA3 and CA1 regions during memory task performance. Proc Nat Acad Sci USA. (2007) 104:14495–500. 10.1073/pnas.0701826104 17726109 PMC1964875

[73] Buzsáki G Wang X-J. Mechanisms of gamma oscillations. Annu Rev Neurosci. (2012) 35:203–25. 10.1146/annurev-neuro-062111-150444 22443509 PMC4049541

[74] Bartos M Vida I Jonas P. Synaptic mechanisms of synchronized gamma oscillations in inhibitory interneuron networks. Nat Rev Neurosci. (2007) 8:45–56. 10.1038/nrn2044 17180162

[75] Nuñez A Buño W. The theta rhythm of the hippocampus: from neuronal and circuit mechanisms to behavior. Front Cell Neurosci. (2021) 15:649262. 10.3389/fncel.2021.649262 33746716 PMC7970048

[76] Tukker JJ Lasztoczi B Katona L Roberts JDB Pissadaki EK Dalezios Y. Distinct dendritic arborization and in vivo firing patterns of parvalbumin-expressing basket cells in the hippocampal area CA3. J Neurosci. (2013) 33:6809–25. 10.1523/JNEUROSCI.5052-12.2013 23595740 PMC4473055

[77] Harris KD Henze DA Hirase H Leinekugel X Dragoi G Czurkó A. Spike train dynamics predicts theta-related phase precession in hippocampal pyramidal cells. Nature. (2002) 417:738–41. 10.1038/nature00808 12066184

[78] Klausberger T Somogyi P. Neuronal diversity and temporal dynamics: the unity of hippocampal circuit operations. Science. (2008) 321:53–7. 10.1126/science.1149381 18599766 PMC4487503

[79] O'Keefe J Recce ML. Phase relationship between hippocampal place units and the EEG theta rhythm. Hippocampus. (1993) 3:317–30. 10.1002/hipo.450030307 8353611

[80] Skaggs WE McNaughton BL Wilson MA Barnes CA. Theta phase precession in hippocampal neuronal populations and the compression of temporal sequences. Hippocampus. (1996) 6:149–72. 10.1002/(SICI)1098-1063(1996)6:2<149::AID-HIPO6>3.0.CO;2-K 8797016

[81] Magee JC Johnston D. A synaptically controlled, associative signal for hebbian plasticity in hippocampal neurons. Science. (1997) 275:209–13. 10.1126/science.275.5297.209 8985013

[82] Umbach G Kantak P Jacobs J Kahana M Pfeiffer BE Sperling M. Time cells in the human hippocampus and entorhinal cortex support episodic memory. Proc Nat Acad Sci USA. (2020) 117:28463–74. 10.1073/pnas.2013250117 33109718 PMC7668099

[83] Fries P. A mechanism for cognitive dynamics: neuronal communication through neuronal coherence. Trends Cogn Sci. (2005) 9:474–80. 10.1016/j.tics.2005.08.011 16150631

[84] Buzsáki G. Neural syntax: cell assemblies, synapsembles, and readers. Neuron. (2010) 68:362–85. 10.1016/j.neuron.2010.09.023 21040841 PMC3005627

[85] DeCoteau WE Thorn C Gibson DJ Courtemanche R Mitra P Kubota Y. Learning-related coordination of striatal and hippocampal theta rhythms during acquisition of a procedural maze task. Proc Nat Acad Sci USA. (2007) 104:5644–9. 10.1073/pnas.0700818104 17372196 PMC1838454

[86] Gregoriou GG Gotts SJ Zhou H Desimone R. High-frequency, long-range coupling between prefrontal and visual cortex during attention. Science. (2009) 324:1207–10. 10.1126/science.1171402 19478185 PMC2849291

[87] Colgin LL Denninger T Fyhn M Hafting T Bonnevie T Jensen O. Frequency of gamma oscillations routes flow of information in the hippocampus. Nature. (2009) 462:353–7. 10.1038/nature08573 19924214

[88] Fell J Klaver P Lehnertz K Grunwald T Schaller C Elger CE. Human memory formation is accompanied by rhinal–hippocampal coupling and decoupling. Nat Neurosci. (2001) 4:1259–64. 10.1038/nn759 11694886

[89] Siegel M Donner TH Engel AK. Spectral fingerprints of large-scale neuronal interactions. Nat Rev Neurosci. (2012) 13:121–34. 10.1038/nrn3137 22233726

[90] Spencer KM Nestor PG Niznikiewicz MA Salisbury DF Shenton ME McCarley RW. Abnormal neural synchrony in schizophrenia. J Neurosci. (2003) 23:7407–11. 10.1523/JNEUROSCI.23-19-07407.2003 12917376 PMC2848257

[91] Herrmann C Demiralp T. Human EEG gamma oscillations in neuropsychiatric disorders. Clin Neurophysiol. (2005) 116:2719–33. 10.1016/j.clinph.2005.07.007 16253555

[92] Lega BC Jacobs J Kahana M. Human hippocampal theta oscillations and the formation of episodic memories. Hippocampus. (2012) 22:748–61. 10.1002/hipo.20937 21538660

[93] Barry RJ Clarke AR. Resting state brain oscillations and symptom profiles in attention deficit/hyperactivity disorder. Clin Neurophysiol. (2013) 17:275–87. 10.1016/B978-0-7020-5307-8.00017-X 24053045

[94] Inostroza M Brotons-Mas JR Laurent F Cid E de la Prida LM. Specific impairment of “what-where-when” episodic-like memory in experimental models of temporal lobe epilepsy. J Neurosci. (2013) 33:17749–62. 10.1523/JNEUROSCI.0957-13.2013 24198366 PMC6618429

[95] Wang J Fang Y Wang X Yang H Yu X Wang H. Enhanced gamma activity and cross-frequency interaction of resting-state electroencephalographic oscillations in patients with Alzheimer's disease. Front Aging Neurosci. (2017) 9:243. 10.3389/fnagi.2017.00243 28798683 PMC5526997

[96] Purves D Williams SM. Neuroscience. 2nd ed. Sunderland, MA: Sinauer Associates (2001).

[97] Citri A Malenka RC. Synaptic plasticity: multiple forms, functions, and mechanisms. Neuropsychopharmacology. (2008) 33:18–41. 10.1038/sj.npp.1301559 17728696

[98] Rotenberg A Mayford M Hawkins RD Kandel ER Muller RU. Mice expressing activated CaMKII lack low frequency LTP and do not form stable place cells in the CA1 region of the hippocampus. Cell. (1996) 87:1351–61. 10.1016/S0092-8674(00)81829-2 8980240

[99] Kentros C Hargreaves E Hawkins RD Kandel ER Shapiro M Muller RV. Abolition of long-term stability of new hippocampal place cell maps by NMDA receptor blockade. Science. (1998) 280:2121–6. 10.1126/science.280.5372.2121 9641919

[100] Barry JM Rivard B Fox SE Fenton AA Sacktor TC Muller RU. Inhibition of protein kinase M disrupts the stable spatial discharge of hippocampal place cells in a familiar environment. J Neurosci. (2012) 32:13753–62. 10.1523/JNEUROSCI.0319-12.2012 23035087 PMC3752127

[101] Schoenenberger P O'Neill J Csicsvari J. Activity-dependent plasticity of hippocampal place maps. Nat Commun. (2016) 7:11824. 10.1038/ncomms11824 27282121 PMC4906387

[102] Knierim JJ. Synaptic plasticity and place cell formation. Encycl Neurosci. (2009) 5:735–40. 10.1016/B978-008045046-9.00830-5

[103] Derdikman D Moser EI. A manifold of spatial maps in the brain. Space Time Number Brain. (2011) 9:41–57. 10.1016/B978-0-12-385948-8.00004-9

[104] Ashby DM Floresco SB Phillips AG McGirr A Seamans JK Wang YT. LTD is involved in the formation and maintenance of rat hippocampal CA1 place-cell fields. Nat Commun. (2021) 12:100. 10.1038/s41467-020-20317-7 33397954 PMC7782827

[105] Sun L Liu SY Zhou XW Wang XC Liu R Wang Q. Inhibition of protein phosphatase 2A- and protein phosphatase 1-induced tau hyperphosphorylation and impairment of spatial memory retention in rats. Neuroscience. (2003) 118:1175–82. 10.1016/S0306-4522(02)00697-8 12732260

[106] Ge Y Dong Z Bagot RC Howland JG Phillips AG Wong TP. Hippocampal long-term depression is required for the consolidation of spatial memory. Proc Nat Acad Sci USA. (2010) 107:16697–702. 10.1073/pnas.1008200107 20823230 PMC2944752

[107] Blackman AV Abrahamsson T Costa RP Lalanne T Sjöström PJ. Target-cell-specific short-term plasticity in local circuits. Front Synaptic Neurosci. (2013) 5:11. 10.3389/fnsyn.2013.00011 24367330 PMC3854841

[108] Tsodyks M Uziel A Markram H. Synchrony generation in recurrent networks with frequency-dependent synapses. J Neurosci. (2000) 20:RC50. 10.1523/JNEUROSCI.20-01-j0003.2000 10627627 PMC6774142

[109] Deng P-Y Klyachko VA. The diverse functions of short-term plasticity components in synaptic computations. Commun Integr Biol. (2011) 4:543–8. 10.4161/cib.15870 22046457 PMC3204123

[110] Austin JK Dunn DW Caffrey HM Perkins SM Harezlak J Rose DF. Recurrent seizures and behavior problems in children with first recognized seizures: a prospective study. Epilepsia. (2002) 43:1564–73. 10.1046/j.1528-1157.2002.26002.x 12460260

[111] Oostrom KJ Smeets-Schouten A Kruitwagen CLJJ Peters ACB Jennekens-Schinkel A for for the Dutch Study Group of Epilepsy in Childhood. Not only a matter of epilepsy: early problems of cognition and behavior in children with “epilepsy only”—a prospective, longitudinal, controlled study starting at diagnosis. Pediatrics. (2003) 112:1338–44. 10.1542/peds.112.6.1338 14654607

[112] Keezer MR Sisodiya SM Sander JW. Comorbidities of epilepsy: current concepts and future perspectives. Lancet Neurol. (2016) 15:106–15. 10.1016/S1474-4422(15)00225-2 26549780

[113] Elger CE Helmstaedter C Kurthen M. Chronic epilepsy and cognition. Lancet Neurol. (2004) 3:663–72. 10.1016/S1474-4422(04)00906-8 15488459

[114] Berg AT Smith SN Frobish D Levy SR Testa FM Beckerman B. Special education needs of children with newly diagnosed epilepsy. Dev Med Child Neurol. (2005) 47:749. 10.1017/S001216220500157X 16225738

[115] Pulliainen V Kuikka P Jokelainen M. Motor and cognitive functions in newly diagnosed adult seizure patients before antiepileptic medication: cognitive functions in newly diagnosed seizure patients. Acta Neurol Scand. (2000) 101:73–8. 10.1034/j.1600-0404.2000.101002073.x 10685851

[116] Lawson JA Vogrin S Bleasel AF Cook MJ Burns L McAnally L. Predictors of hippocampal, cerebral, and cerebellar volume reduction in childhood epilepsy. Epilepsia. (2000) 41:1540–5. 10.1111/j.1528-1157.2000.tb00122.x 11114211

[117] Keller SS Roberts N. Voxel-based morphometry of temporal lobe epilepsy: an introduction and review of the literature. Epilepsia. (2008) 49:741–57. 10.1111/j.1528-1167.2007.01485.x 18177358

[118] Seidenberg M O'leary DS Giordani B Berent S Boll TJ. Test-retest IQ changes of epilepsy patients: assessing the influence of practice effects. J Clin Neuropsychol. (1981) 3:237–55. 10.1080/01688638108403128 7328177

[119] Oyegbile TO Dow C Jones J Bell B Rutecki P Sheth R. The nature and course of neuropsychological morbidity in chronic temporal lobe epilepsy. Neurology. (2004) 62:1736–42. 10.1212/01.WNL.0000125186.04867.34 15159470

[120] Rayner G Jackson GD Wilson SJ. Mechanisms of memory impairment in epilepsy depend on age at disease onset. Neurology. (2016) 87:1642–9. 10.1212/WNL.0000000000003231 27638925 PMC5085077

[121] Clawson W Madec T Ghestem A Quilichini PP Battaglia D Bernard C. Disordered information processing dynamics in experimental epilepsy. preprint. Neuroscience. (2021) 2021:430768. 10.1101/2021.02.11.430768 PMC10513075 37550052

[122] Liu X Muller RU Huang L-T Kubie JL Rotenberg A Rivard B. Seizure-induced changes in place cell physiology: relationship to spatial memory. J Neurosci. (2003) 23:11505–15. 10.1523/JNEUROSCI.23-37-11505.2003 14684854 PMC6740937

[123] Ewell LA Fischer KB Leibold C Leutgeb S Leutgeb JK. The impact of pathological high-frequency oscillations on hippocampal network activity in rats with chronic epilepsy. Elife. (2019) 8:e42148. 10.7554/eLife.42148 30794155 PMC6386518

[124] Shuman T Aharoni D Cai DJ Lee CR Chavlis S Page-Harley L. Breakdown of spatial coding and interneuron synchronization in epileptic mice. Nat Neurosci. (2020) 23:229–38. 10.1038/s41593-019-0559-0 31907437 PMC7259114

[125] Chauviere L Rafrafi N Thinus-Blanc C Bartolomei F Esclapez M Bernard C. Early deficits in spatial memory and theta rhythm in experimental temporal lobe epilepsy. J Neurosci. (2009) 29:5402–10. 10.1523/JNEUROSCI.4699-08.2009 19403808 PMC6665868

[126] Karnam HB Zhou J-L Huang L-T Zhao Q Shatskikh T Holmes GL. Early life seizures cause long-standing impairment of the hippocampal map. Exp Neurol. (2009) 217:378–87. 10.1016/j.expneurol.2009.03.028 19345685 PMC2791529

[127] Brun VH Leutgeb S Wu H-Q Schwarcz R Witter MP Moser EI. Impaired spatial representation in CA1 after lesion of direct input from entorhinal cortex. Neuron. (2008) 57:290–302. 10.1016/j.neuron.2007.11.034 18215625

[128] Davoudi H Foster DJ. Acute silencing of hippocampal CA3 reveals a dominant role in place field responses. Nat Neurosci. (2019) 22:337–42. 10.1038/s41593-018-0321-z 30664772 PMC6387637

[129] Tyler AL Mahoney JM Richard GR Holmes GL Lenck-Santini P-P Scott RC. Functional network changes in hippocampal CA1 after status epilepticus predict spatial memory deficits in rats. J Neurosci. (2012) 32:11365–76. 10.1523/JNEUROSCI.1516-12.2012 22895719 PMC3536550

[130] Yu JY Frank LM. Hippocampal–cortical interaction in decision making. Neurobiol Learn Mem. (2015) 117:34–41. 10.1016/j.nlm.2014.02.002 24530374 PMC4133322

[131] Hernan A Mahoney J Curry W Mawe S Scott R. Distributed dynamic coding for spatial working memory in hippocampal-prefrontal networks. Neuroscience. (2019) 2019:630673. 10.1101/630673

[132] Nash MI Hodges CB Muncy NM Kirwan CB. Pattern separation beyond the hippocampus: a high-resolution whole-brain investigation of mnemonic discrimination in healthy adults. Hippocampus. (2021) 31:408–21. 10.1002/hipo.23299 33432734

[133] Bakker A Krauss GL Albert MS Speck CL Jones LR Stark CE. Reduction of hippocampal hyperactivity improves cognition in amnestic mild cognitive impairment. Neuron. (2012) 74:467–74. 10.1016/j.neuron.2012.03.023 22578498 PMC3351697

[134] Brock Kirwan C Hartshorn A Stark SM Goodrich-Hunsaker NJ Hopkins RO Stark CEL. Pattern separation deficits following damage to the hippocampus. Neuropsychologia. (2012) 50:2408–14. 10.1016/j.neuropsychologia.2012.06.011 22732491 PMC3411917

[135] Yassa MA Stark SM Bakker A Albert MS Gallagher M Stark CEL. High-resolution structural and functional MRI of hippocampal CA3 and dentate gyrus in patients with amnestic Mild Cognitive Impairment. Neuroimage. (2010) 51:1242–52. 10.1016/j.neuroimage.2010.03.040 20338246 PMC2909476

[136] Bui AD Nguyen TM Limouse C Kim HK Szabo GG Felong S. Dentate gyrus mossy cells control spontaneous convulsive seizures and spatial memory. Science. (2018) 359:787–90. 10.1126/science.aan4074 29449490 PMC6040648

[137] Bernhardt BC Bonilha L Gross DW. Network analysis for a network disorder: the emerging role of graph theory in the study of epilepsy. Epilepsy Behav. (2015) 50:162–70. 10.1016/j.yebeh.2015.06.005 26159729

[138] Yilmazer-Hanke DM Wolf HK Schramm J Elger CE Wiestler OD Blümcke I. Subregional pathology of the amygdala complex and entorhinal region in surgical specimens from patients with pharmacoresistant temporal lobe epilepsy. J Neuropathol Exp Neurol. (2000) 59:907–20. 10.1093/jnen/59.10.907 11079781

[139] Bothwell S Meredith GE Phillips J Staunton H Doherty C Grigorenko E. Neuronal hypertrophy in the neocortex of patients with temporal lobe epilepsy. J Neurosci. (2001) 21:4789–800. 10.1523/JNEUROSCI.21-13-04789.2001 11425906 PMC6762344

[140] Blanc F Martinian L Liagkouras I Catarino C Sisodiya SM Thom M. Investigation of widespread neocortical pathology associated with hippocampal sclerosis in epilepsy: a postmortem study. Epilepsia. (2011) 52:10–21. 10.1111/j.1528-1167.2010.02773.x 21198557

[141] Sinjab B Martinian L Sisodiya SM Thom M. Regional thalamic neuropathology in patients with hippocampal sclerosis and epilepsy: a postmortem study. Epilepsia. (2013) 54:2125–33. 10.1111/epi.12403 24138281 PMC3995016

[142] Tai XY Bernhardt B Thom M Thompson P Baxendale S Koepp M. Review: neurodegenerative processes in temporal lobe epilepsy with hippocampal sclerosis: clinical, pathological and neuroimaging evidence. Neuropathol Appl Neurobiol. (2018) 44:70–90. 10.1111/nan.12458 29288503

[143] Cendes F Andermann F Gloor P Evans A Jones-Gotman M Watson C. MRI volumetric measurement of amygdala and hippocampus in temporal lobe epilepsy. Neurology. (1993) 43:719–719. 10.1212/WNL.43.4.719 8469329

[144] Bernasconi N Bernasconi A Caramanos Z Dubeau F Richardson J Andermann F. Entorhinal cortex atrophy in epilepsy patients exhibiting normal hippocampal volumes. Neurology. (2001) 56:1335–9. 10.1212/WNL.56.10.1335 11376184

[145] Bernasconi N Bernasconi A Caramanos Z Antel SB Andermann F Arnold DL. Mesial temporal damage in temporal lobe epilepsy: a volumetric MRI study of the hippocampus, amygdala and parahippocampal region. Brain. (2003) 126:462–9. 10.1093/brain/awg034 12538412

[146] Bernhardt BC Hong S-J Bernasconi A Bernasconi N. Magnetic resonance imaging pattern learning in temporal lobe epilepsy: classification and prognostics. Ann Neurol. (2015) 77:436–46. 10.1002/ana.24341 25546153

[147] Bernhardt BC Worsley KJ Kim H Evans AC Bernasconi A Bernasconi N. Longitudinal and cross-sectional analysis of atrophy in pharmacoresistant temporal lobe epilepsy. Neurology. (2009) 72:1747–54. 10.1212/01.wnl.0000345969.57574.f5 19246420 PMC2827310

[148] Coan AC Appenzeller S Bonilha L Li LM Cendes F. Seizure frequency and lateralization affect progression of atrophy in temporal lobe epilepsy. Neurology. (2009) 73:834–42. 10.1212/WNL.0b013e3181b783dd 19752449

[149] Bernhardt BC Kim H Bernasconi N. Patterns of subregional mesiotemporal disease progression in temporal lobe epilepsy. Neurology. (2013) 81:1840–7. 10.1212/01.wnl.0000436069.20513.92 24142475 PMC3821710

[150] Concha L Beaulieu C Gross DW. Bilateral limbic diffusion abnormalities in unilateral temporal lobe epilepsy. Ann Neurol. (2005) 57:188–96. 10.1002/ana.20334 15562425

[151] Yogarajah M Duncan JS. Diffusion-based magnetic resonance imaging and tractography in epilepsy. Epilepsia. (2008) 49:189–200. 10.1111/j.1528-1167.2007.01378.x 17941849

[152] Bonilha L Edwards JC Kinsman SL Morgan PS Fridriksson J Rorden C. Extrahippocampal gray matter loss and hippocampal deafferentation in patients with temporal lobe epilepsy. Epilepsia. (2010) 51:519–28. 10.1111/j.1528-1167.2009.02506.x 20163442 PMC2855766

[153] Hermann B Conant LL Cook CJ Hwang G Garcia-Ramos C Dabbs K. Network, clinical and sociodemographic features of cognitive phenotypes in temporal lobe epilepsy. NeuroImage. (2020) 27:102341. 10.1016/j.nicl.2020.102341 32707534 PMC7381697

[154] Bernhardt BC Worsley KJ Besson P Concha L Lerch JP Evans AC. Mapping limbic network organization in temporal lobe epilepsy using morphometric correlations: insights on the relation between mesiotemporal connectivity and cortical atrophy. Neuroimage. (2008) 42:515–24. 10.1016/j.neuroimage.2008.04.261 18554926

[155] Mueller SG Laxer KD Barakos J Cheong I Garcia P Weiner MW. Widespread neocortical abnormalities in temporal lobe epilepsy with and without mesial sclerosis. Neuroimage. (2009) 46:353–9. 10.1016/j.neuroimage.2009.02.020 19249372 PMC2799165

[156] Mueller SG Laxer KD Barakos J Cheong I Finlay D Garcia P. Involvement of the thalamocortical network in TLE with and without mesiotemporal sclerosis: thalamocortical Network in TLE. Epilepsia. (2009) 51:1436–45. 10.1111/j.1528-1167.2009.02413.x 20002143 PMC2888933

[157] Bettus G Bartolomei F Confort-Gouny S Guedj E Chauvel P Cozzone PJ. Role of resting state functional connectivity MRI in presurgical investigation of mesial temporal lobe epilepsy. J Neurol Neurosurg Psychiatry. (2010) 81:1147–54. 10.1136/jnnp.2009.191460 20547611

[158] Pereira FRS Alessio A Sercheli MS Pedro T Bilevicius E Rondina JM. Asymmetrical hippocampal connectivity in mesial temporal lobe epilepsy: evidence from resting state fMRI. BMC Neurosci. (2010) 11:66. 10.1186/1471-2202-11-66 20525202 PMC2890013

[159] Zhang C Yang H Qin W Liu C Qi Z Chen N. Characteristics of resting-state functional connectivity in intractable unilateral temporal lobe epilepsy patients with impaired executive control function. Front Hum Neurosci. (2017) 11:609. 10.3389/fnhum.2017.00609 29375338 PMC5770650

[160] Zhang Z Lu G Zhong Y Tan Q Liao W Wang Z. Altered spontaneous neuronal activity of the default-mode network in mesial temporal lobe epilepsy. Brain Res. (2010) 1323:152–60. 10.1016/j.brainres.2010.01.042 20132802

[161] Liao W Zhang Z Pan Z Mantini D Ding J Duan X. Default mode network abnormalities in mesial temporal lobe epilepsy: a study combining fMRI and DTI. Hum Brain Mapp. (2011) 32:883–95. 10.1002/hbm.21076 20533558 PMC6870458

[162] Pittau F Grova C Moeller F Dubeau F Gotman J. Patterns of altered functional connectivity in mesial temporal lobe epilepsy: functional connectivity in MTLE. Epilepsia. (2012) 53:1013–23. 10.1111/j.1528-1167.2012.03464.x 22578020 PMC3767602

[163] Haneef Z Lenartowicz A Yeh HJ Levin HS Engel J Stern JM. Functional connectivity of hippocampal networks in temporal lobe epilepsy. Epilepsia. (2014) 55:137–45. 10.1111/epi.12476 24313597 PMC3946924

[164] Zhang C Zhang H Xu K Yang H Liu C Yu T. Impaired prefrontal cortex-thalamus pathway in intractable temporal lobe epilepsy with aberrant executive control function: MRI evidence. Clin Neurophysiol. (2019) 130:484–90. 10.1016/j.clinph.2018.12.007 30771725

[165] Bettus G Guedj E Joyeux F Confort-Gouny S Soulier E Laguitton V. Decreased basal fMRI functional connectivity in epileptogenic networks and contralateral compensatory mechanisms. Hum Brain Mapp. (2009) 30:1580–91. 10.1002/hbm.20625 18661506 PMC6870867

[166] Morgan VL Rogers BP Sonmezturk HH Gore JC Abou-Khalil B. Cross hippocampal influence in mesial temporal lobe epilepsy measured with high temporal resolution functional magnetic resonance imaging. Epilepsia. (2011) 52:1741–9. 10.1111/j.1528-1167.2011.03196.x 21801166 PMC4428312

[167] Bai X Guo J Killory B Vestal M Berman R Negishi M. Resting functional connectivity between the hemispheres in childhood absence epilepsy. Neurology. (2011) 76:1960–7. 10.1212/WNL.0b013e31821e54de 21646622 PMC3109878

[168] Luo C Li Q Xia Y Lei X Xue K Yao Z. Resting state basal ganglia network in idiopathic generalized epilepsy. Hum Brain Mapp. (2012) 33:1279–94. 10.1002/hbm.21286 21520351 PMC6869872

[169] Masterton RA Carney PW Jackson GD. Cortical and thalamic resting-state functional connectivity is altered in childhood absence epilepsy. Epilepsy Res. (2012) 99:327–34. 10.1016/j.eplepsyres.2011.12.014 22281064

[170] McGill ML Devinsky O Kelly C Milham M Castellanos FX Quinn BT. Default mode network abnormalities in idiopathic generalized epilepsy. Epilepsy Behav. (2012) 23:353–9. 10.1016/j.yebeh.2012.01.013 22381387 PMC4407647

[171] Yang T Luo C Li Q Guo Z Liu L Gong Q. Altered resting-state connectivity during interictal generalized spike-wave discharges in drug-naïve childhood absence epilepsy. Hum Brain Mapp. (2013) 34:1761–7. 10.1002/hbm.22025 22431250 PMC6870260

[172] Diessen E van Zweiphenning WJEM Jansen FE Stam CJ Braun KPJ Otte WM. Brain network organization in focal epilepsy: a systematic review and meta-analysis. PLoS ONE. (2014) 9:e114606. 10.1371/journal.pone.0114606 25493432 PMC4262431

[173] Vlooswijk MCG Vaessen MJ Jansen JFA de Krom MCFTM Majoie HJM Hofman P. Loss of network efficiency associated with cognitive decline in chronic epilepsy. Neurology. (2011) 77:938–44. 10.1212/WNL.0b013e31822cfc2f 21832213

[174] Vaessen MJ Jansen JFA Vlooswijk MCG Hofman PAM Majoie HJM Aldenkamp AP. White matter network abnormalities are associated with cognitive decline in chronic epilepsy. Cerebr Cortex. (2012) 22:2139–47. 10.1093/cercor/bhr298 22038907

[175] Gauffin H van Ettinger-Veenstra H Landtblom A-M Ulrici D McAllister A Karlsson T. Impaired language function in generalized epilepsy: inadequate suppression of the default mode network. Epilepsy Behav. (2013) 28:26–35. 10.1016/j.yebeh.2013.04.001 23648277

[176] Hampson M Driesen NR Skudlarski P Gore JC Constable RT. Brain connectivity related to working memory performance. J Neurosci. (2006) 26:13338–43. 10.1523/JNEUROSCI.3408-06.2006 17182784 PMC2677699

[177] Kelly AMC Uddin LQ Biswal BB Castellanos FX Milham MP. Competition between functional brain networks mediates behavioral variability. Neuroimage. (2008) 39:527–37. 10.1016/j.neuroimage.2007.08.008 17919929

[178] Bernhardt BC Chen Z He Y Evans AC Bernasconi N. Graph-theoretical analysis reveals disrupted small-world organization of cortical thickness correlation networks in temporal lobe epilepsy. Cerebr Cortex. (2011) 21:2147–57. 10.1093/cercor/bhq291 21330467

[179] Diessen E van Diederen SJH Braun KPJ Jansen FE Stam CJ. Functional and structural brain networks in epilepsy: what have we learned? Epilepsia. (2013) 54:1855–65. 10.1111/epi.12350 24032627

[180] Rayner G Tailby C Jackson G Wilson S. Looking beyond lesions for causes of neuropsychological impairment in epilepsy. Neurology. (2019) 92:e680–9. 10.1212/WNL.0000000000006905 30635484 PMC6382365

[181] Sakkaki S Barrière S Bender AC Scott RC Lenck-Santini PP. Focal dorsal hippocampal Nav1.1 knock down alters place cell temporal coordination and spatial behavior. Cerebr Cortex. (2020) 30:5049–66. 10.1093/cercor/bhaa101 32377688 PMC8475810

[182] Bender AC Luikart BW Lenck-Santini PP. Cognitive deficits associated with Nav1.1 alterations: involvement of neuronal firing dynamics and oscillations. PLoS ONE. (2016) 11:e0151538. 10.1371/journal.pone.0151538 26978272 PMC4792481

[183] Lucas MM Lenck-Santini P-P Holmes GL Scott RC. Impaired cognition in rats with cortical dysplasia: additional impact of early-life seizures. Brain. (2011) 134:1684–93. 10.1093/brain/awr087 21602270 PMC3102240

[184] Weglage J Demsky A Pietsch M Kurlemann G. Neuropsychological, intellectual, and behavioral findings in patients with centrotemporal spikes with and without seizures. Dev Med Child Neurol. (1997) 39:646–51. 10.1111/j.1469-8749.1997.tb07357.x 9352724

[185] Fonseca LC Tedrus GMAS Tonelotto JM Antunes T Chiodi MG. School performance in children with benign childhood epilepsy with centrotemporal spikes. Arquivos De Neuro-Psiquiatria. (2004) 62:459–62. 10.1590/S0004-282X2004000300015 15273844

[186] Kleen JK Scott RC Holmes GL Lenck-Santini PP. Hippocampal interictal spikes disrupt cognition in rats. Ann Neurol. (2010) 67:250–7. 10.1002/ana.21896 20225290 PMC2926932

[187] Kleen JK Scott RC Holmes GL Roberts DW Rundle MM Testorf M. Hippocampal interictal epileptiform activity disrupts cognition in humans. Neurology. (2013) 81:18–24. 10.1212/WNL.0b013e318297ee50 23685931 PMC3770206

[188] Suárez LM Cid E Gal B Inostroza M Brotons-Mas JR Gómez-Domínguez D. Systemic injection of kainic acid differently affects LTP magnitude depending on its epileptogenic efficiency. PLoS ONE. (2012) 7:e48128. 10.1371/journal.pone.0048128 23118939 PMC3485282

[189] Lenz M Ben Shimon M Deller T Vlachos A Maggio N. Pilocarpine-induced status epilepticus is associated with changes in the actin-modulating protein synaptopodin and alterations in long-term potentiation in the mouse hippocampus. Neural Plast. (2017) 2017:1–7. 10.1155/2017/2652560 28154762 PMC5244022

[190] Lynch M Sayin Ü Bownds J Janumpalli S Sutula T. Long-term consequences of early postnatal seizures on hippocampal learning and plasticity: developmental plasticity in the hippocampus. Eur J Neurosci. (2000) 12:2252–64. 10.1046/j.1460-9568.2000.00117.x 10947804

[191] Hernan AE Holmes GL Isaev D Scott RC Isaeva E. Altered short-term plasticity in the prefrontal cortex after early life seizures. Neurobiol Dis. (2013) 50:120–6. 10.1016/j.nbd.2012.10.007 23064435 PMC3534893

[192] Hernan AE Alexander A Jenks KR Barry J Lenck-Santini P-P Isaeva E. Focal epileptiform activity in the prefrontal cortex is associated with long-term attention and sociability deficits. Neurobiol Dis. (2014) 63:25–34. 10.1016/j.nbd.2013.11.012 24269731 PMC4397918

[193] Schlesiger MI Cannova CC Boublil BL Hales JB Mankin EA Brandon MP. The medial entorhinal cortex is necessary for temporal organization of hippocampal neuronal activity. Nat Neurosci. (2015) 18:1123–32. 10.1038/nn.4056 26120964 PMC4711275

[194] Hales JB Schlesiger MI Leutgeb JK Squire LR Leutgeb S Clark RE. Medial entorhinal cortex lesions only partially disrupt hippocampal place cells and hippocampus-dependent place memory. Cell Rep. (2014) 9:893–901. 10.1016/j.celrep.2014.10.009 25437546 PMC4294707

[195] Lenck-Santini P-P Holmes GL. Altered phase precession and compression of temporal sequences by place cells in epileptic rats. J Neurosci. (2008) 28:5053–62. 10.1523/JNEUROSCI.5024-07.2008 18463258 PMC3304586

[196] Powers S Miranda C Dennys-Rivers C Huffenberger A Braun L Rinaldi F. Rett syndrome gene therapy improves survival and ameliorates behavioral phenotypes in MeCP2 null (S51.002). Neurology. (2019) 92(15Suppl.):S51.002.

[197] Moretti P M. Levenson J Battaglia F Atkinson R Teague R. Learning and memory and synaptic plasticity are impaired in a mouse model of Rett syndrome. J Neurosci. (2006) 26:319–27. 10.1523/JNEUROSCI.2623-05.2006 16399702 PMC6674314

[198] Hagberg B Aicardi J Dias K Ramos O. A progressive syndrome of autism, dementia, ataxia, and loss of purposeful hand use in girls: Rett's syndrome: report of 35 cases. Ann Neurol. (1983) 14:471–9. 10.1002/ana.410140412 6638958

[199] Amir RE Van den Veyver IB Wan M Tran CQ Francke U Zoghbi HY. Rett syndrome is caused by mutations in X-linked MECP2, encoding methyl-CpG-binding protein 2. Nat Genet. (1999) 23:185–8. 10.1038/13810 10508514

[200] Chahrour M Jung SY Shaw C Zhou X Wong STC Qin J. MeCP2, a key contributor to neurological disease, activates and represses transcription. Science. (2008) 320:1224–9. 10.1126/science.1153252 18511691 PMC2443785

[201] Moretti P Bouwknecht JA Teague R Paylor R Zoghbi HY. Abnormalities of social interactions and home-cage behavior in a mouse model of Rett syndrome. Hum Mol Genet. (2005) 14:205–20. 10.1093/hmg/ddi016 15548546

[202] Stearns NA Schaevitz LR Bowling H Nag N Berger UV Berger-Sweeney J. Behavioral and anatomical abnormalities in Mecp2 mutant mice: a model for Rett syndrome. Neuroscience. (2007) 146:907–21. 10.1016/j.neuroscience.2007.02.009 17383101

[203] Kee SE Mou X Zoghbi HY Ji D. Impaired spatial memory codes in a mouse model of Rett syndrome. Elife. (2018) 7:e31451. 10.7554/eLife.31451 30028675 PMC6054527

[204] Asaka Y Jugloff DGM Zhang L Eubanks JH Fitzsimonds RM. Hippocampal synaptic plasticity is impaired in the Mecp2-null mouse model of Rett syndrome. Neurobiol Dis. (2006) 21:217–27. 10.1016/j.nbd.2005.07.005 16087343

[205] Cardoza B Clarke A Wilcox J Gibbon F Smith PEM Archer H. Epilepsy in Rett syndrome: association between phenotype and genotype, and implications for practice. Seizure. (2011) 20:646–9. 10.1016/j.seizure.2011.06.010 21764336

[206] Ito-Ishida A Ure K Chen H Swann JW Zoghbi HY. Loss of MeCP2 in parvalbumin-and somatostatin-expressing neurons in mice leads to distinct Rett syndrome-like phenotypes. Neuron. (2015) 88:651–8. 10.1016/j.neuron.2015.10.029 26590342 PMC4656196

[207] Operto FF Mazza R Pastorino GMG Verrotti A Coppola G. Epilepsy and genetic in Rett syndrome: a review. Brain Behav. (2019) 9:e01250. 10.1002/brb3.1250 30929312 PMC6520293

[208] Buzsaki G Horvath Z Urioste R Hetke J Wise K. High-frequency network oscillation in the hippocampus. Science. (1992) 256:1025–7. 10.1126/science.1589772 1589772

[209] Buzsáki G. Two-stage model of memory trace formation: a role for “noisy” brain states. Neuroscience. (1989) 31:551–70. 10.1016/0306-4522(89)90423-5 2687720

[210] Lang M Wither RG Brotchie JM Wu C Zhang L Eubanks JH. Selective preservation of MeCP2 in catecholaminergic cells is sufficient to improve the behavioral phenotype of male and female Mecp2-deficient mice. Hum Mol Genet. (2013) 22:358–71. 10.1093/hmg/dds433 23077217

[211] Samarasinghe RA Miranda OA Buth JE Mitchell S Ferando I Watanabe M. Identification of neural oscillations and epileptiform changes in human brain organoids. Nat Neurosci. (2021) 24:1488–500. 10.1038/s41593-021-00906-5 34426698 PMC9070733

[212] D'Cruz JA Wu C Zahid T El-Hayek Y Zhang L Eubanks JH. Alterations of cortical and hippocampal EEG activity in MeCP2-deficient mice. Neurobiol Dis. (2010) 38:8–16. 10.1016/j.nbd.2009.12.018 20045053

[213] Kosai K Kusaga A Isagai T Hirata K Nagano S Murofushi Y. Rett syndrome is reversible and treatable by MeCP2 gene therapy into the striatum in mice. Mol Ther. (2005) 11:S24. 10.1016/j.ymthe.2005.06.086

[214] Luoni M Giannelli S Indrigo MT Niro A Massimino L Iannielli A. Whole brain delivery of an instability-prone Mecp2 transgene improves behavioral and molecular pathological defects in mouse models of Rett syndrome. Elife. (2020) 9:e52629. 10.7554/eLife.52629 32207685 PMC7117907

[215] Gadalla KK Bailey ME Spike RC Ross PD Woodard KT Kalburgi SN. Improved survival and reduced phenotypic severity following AAV9/MECP2 gene transfer to neonatal and juvenile male Mecp2 knockout mice. Mol Ther. (2013) 21:18–30. 10.1038/mt.2012.200 23011033 PMC3536818

[216] Kahn JB Port RG Yue C Takano H Coulter DA. Circuit-based interventions in the dentate gyrus rescue epilepsy-associated cognitive dysfunction. Brain. (2019) 142:2705–21. 10.1093/brain/awz209 31363737 PMC6736326

[217] Snowball A Chabrol E Wykes RC Shekh-Ahmad T Cornford JH Lieb A. Epilepsy gene therapy using an engineered potassium channel. J Neurosci. (2019) 39:3159–69. 10.1523/JNEUROSCI.1143-18.2019 30755487 PMC6468110

[218] Hermann B Seidenberg M Lee E-J Chan F Rutecki P. Cognitive phenotypes in temporal lobe epilepsy. J Int Neuropsychol Soc. (2007) 13:12–20. 10.1017/S135561770707004X 17166299

[219] Warburton A Miyajima F Shazadi K Crossley J Johnson MR Marson AG. NRSF and BDNF polymorphisms as biomarkers of cognitive dysfunction in adults with newly diagnosed epilepsy. Epilepsy Behav. (2016) 54:117–27. 10.1016/j.yebeh.2015.11.013 26708060 PMC4732989

[220] Colliva C Ferrari M Benatti C Guerra A Tascedda F Blom JMC. Executive functioning in children with epilepsy: genes matter. Epilepsy Behav. (2019) 95:137–47. 10.1016/j.yebeh.2019.02.019 31054523

[221] Busch RM Lineweaver TT Naugle RI Kim KH Gong Y Tilelli CQ. ApoE-ε4 is associated with reduced memory in long-standing intractable temporal lobe epilepsy. Neurology. (2007) 68:409–14. 10.1212/01.wnl.0000253021.60887.db 17283313

[222] Sidhu MK Thompson PJ Wandschneider B Foulkes A de Tisi J Stretton J. The impact of brain-derived neurotrophic factor Val66Met polymorphism on cognition and functional brain networks in patients with intractable partial epilepsy. CNS Neurosci Ther. (2019) 25:223–32. 10.1111/cns.13003 29952080 PMC6488925

[223] Doherty C Hogue O Floden DP Altemus JB Najm IM Eng C. BDNF and COMT, but not APOE, alleles are associated with psychiatric symptoms in refractory epilepsy. Epilepsy Behav. (2019) 94:131–6. 10.1016/j.yebeh.2019.02.032 30909076 PMC8299517

[224] Hebb D. Condensed program. Am Psychol. (1947) 2:255–352. 10.1037/h0063667

[225] Rosenzweig MR Bennett EL. Cerebral changes in rats exposed individually to an enriched environment. J Comparat Physiol Psychol. (1972) 80:304–13. 10.1037/h0032978 5047833

[226] Nithianantharajah J. Environmental enrichment results in cortical and subcortical changes in levels of synaptophysin and PSD-95 proteins. Neurobiol Learn Mem. (2004) 81:200–10. 10.1016/j.nlm.2004.02.002 15082021

[227] Xu J Sun J Xue Z Li X. An enriched environment reduces the stress level and locomotor activity induced by acute morphine treatment and by saline after chronic morphine treatment in mice. Neuroreport. (2014) 25:701–9. 10.1097/WNR.0000000000000164 24709916

[228] Akyuz E Eroglu E. Envisioning the crosstalk between environmental enrichment and epilepsy: a novel perspective. Epilepsy Behav. (2021) 115:107660. 10.1016/j.yebeh.2020.107660 33328107

[229] Shinohara Y Hosoya A Hirase H. Experience enhances gamma oscillations and interhemispheric asymmetry in the hippocampus. Nat Commun. (2013) 4:1652. 10.1038/ncomms2658 23552067 PMC3644069

[230] Tanaka M Wang X Mikoshiba K Hirase H Shinohara Y. Rearing-environment-dependent hippocampal local field potential differences in wild-type and inositol trisphosphate receptor type 2 knockout mice: experience-dependent hippocampal LFP patterns in WT and IP_3_ R2-KO mice. J Physiol. (2017) 595:6557–68. 10.1113/JP274573 28758690 PMC5638892

[231] Bilkey DK Cheyne KR Eckert MJ Lu X Chowdhury S Worley PF. Exposure to complex environments results in more sparse representations of space in the hippocampus. Hippocampus. (2017) 27:1178–91. 10.1002/hipo.22762 28686801 PMC5752118

[232] Dezsi G Ozturk E Salzberg MR Morris M O'Brien TJ Jones NC. Environmental enrichment imparts disease-modifying and transgenerational effects on genetically-determined epilepsy and anxiety. Neurobiol Dis. (2016) 93:129–36. 10.1016/j.nbd.2016.05.005 27185593

[233] Vrinda M Sasidharan A Aparna S Srikumar BN Kutty BM Shankaranarayana Rao BS. Enriched environment attenuates behavioral seizures and depression in chronic temporal lobe epilepsy. Epilepsia. (2017) 58:1148–58. 10.1111/epi.13767 28480502

[234] Hernan AE Mahoney JM Curry W Richard G Lucas MM Massey A. Environmental enrichment normalizes hippocampal timing coding in a malformed hippocampus. PLoS ONE. (2018) 13:e0191488. 10.1371/journal.pone.0191488 29394267 PMC5796690

[235] The National Institute of Mental Health. Brain Stimulation Therapies. (NIMH). (2016). Available online at: https://www.nimh.nih.gov/health/topics/brain-stimulation-therapies/brain-stimulation-therapies (accessed February 14, 2022).

[236] Turnbull IM McGeer PL Beattie L Calne D Pate B. Stimulation of the basal nucleus of meynert in senile dementia of Alzheimer's type. Stereotact Funct Neurosurg. (1985) 48:216–21. 10.1159/000101130 3915647

[237] Laxton AW Tang-Wai DF McAndrews MP Zumsteg D Wennberg R Keren R. A phase I trial of deep brain stimulation of memory circuits in Alzheimer's disease. Ann Neurol. (2010) 68:521–34. 10.1002/ana.22089 20687206

[238] Sankar T Chakravarty MM Bescos A Lara M Obuchi T Laxton AW. Deep brain stimulation influences brain structure in Alzheimer's disease. Brain Stimul. (2015) 8:645–54. 10.1016/j.brs.2014.11.020 25814404 PMC5659851

[239] Mao Z-Q Wang X Xu X Cui Z-Q Pan L-S Ning X-J. Partial improvement in performance of patients with severe Alzheimer's disease at an early stage of fornix deep brain stimulation. Neural Regen Res. (2018) 13:2164. 10.4103/1673-5374.241468 30323149 PMC6199932

[240] Fontaine D Deudon A Lemaire JJ Razzouk M Viau P Darcourt J. Symptomatic treatment of memory decline in Alzheimer's disease by deep brain stimulation: a feasibility study. J Alzheimer's Dis. (2013) 34:315–23. 10.3233/JAD-121579 23168448

[241] Kuhn J Hardenacke K Shubina E Lenartz D Visser-Vandewalle V Zilles K. Deep brain stimulation of the nucleus basalis of meynert in early stage of Alzheimer's dementia. Brain Stimul. (2015) 8:838–9. 10.1016/j.brs.2015.04.002 25991080

[242] Dürschmid S Reichert C Kuhn J Freund H Hinrichs H Heinze H. Deep brain stimulation of the nucleus basalis of Meynert attenuates early EEG components associated with defective sensory gating in patients with Alzheimer disease – a two-case study. Eur J Neurosci. (2020) 51:1201–9. 10.1111/ejn.13749 29055119

[243] Rossi S Hallett M Rossini PM Pascual-Leone A. Safety, ethical considerations, and application guidelines for the use of transcranial magnetic stimulation in clinical practice and research. Clin Neurophysiol. (2009) 120:2008–39. 10.1016/j.clinph.2009.08.016 19833552 PMC3260536

[244] Cotelli M Manenti R Cappa SF Geroldi C Zanetti O Rossini PM. Effect of transcranial magnetic stimulation on action naming in patients with Alzheimer disease. Arch Neurol. (2006) 63:1602. 10.1001/archneur.63.11.1602 17101829

[245] Cotelli M Manenti R Cappa SF Zanetti O Miniussi C. Transcranial magnetic stimulation improves naming in Alzheimer disease patients at different stages of cognitive decline. Eur J Neurol. (2008) 15:1286–92. 10.1111/j.1468-1331.2008.02202.x 19049544

[246] Bentwich J Dobronevsky E Aichenbaum S Shorer R Peretz R Khaigrekht M. Beneficial effect of repetitive transcranial magnetic stimulation combined with cognitive training for the treatment of Alzheimer's disease: a proof of concept study. J Neural Transm. (2011) 118:463–71. 10.1007/s00702-010-0578-1 21246222

[247] Ahmed MA Darwish ES Khedr EM El Serogy YM Ali AM. Effects of low versus high frequencies of repetitive transcranial magnetic stimulation on cognitive function and cortical excitability in Alzheimer's dementia. J Neurol. (2012) 259:83–92. 10.1007/s00415-011-6128-4 21671144

[248] Gallino D Devenyi GA Germann J Guma E Anastassiadis C Chakravarty MM. Longitudinal assessment of the neuroanatomical consequences of deep brain stimulation: application of fornical DBS in an Alzheimer's mouse model. Brain Res. (2019) 1715:213–23. 10.1016/j.brainres.2019.03.030 30926457

[249] Koulousakis P van den Hove D Visser-Vandewalle V Sesia T. Cognitive improvements after intermittent deep brain stimulation of the nucleus basalis of meynert in a transgenic rat model for Alzheimer's disease: a preliminary approach. J Alzheimer's Dis. (2020) 73:461–6. 10.3233/JAD-190919 31868670

[250] Tsai S-T Chen S-Y Lin S-Z Tseng G-F. Rostral intralaminar thalamic deep brain stimulation ameliorates memory deficits and dendritic regression in β-amyloid-infused rats. Brain Struct Funct. (2020) 225:751–61. 10.1007/s00429-020-02033-6 32036422

[251] Limousin P Krack P Pollak P Benazzouz A Ardouin C Hoffmann D. Electrical stimulation of the subthalamic nucleus in advanced Parkinson's disease. N Engl J Med. (1998) 339:1105–11. 10.1056/NEJM199810153391603 9770557

[252] Volkmann J Sturm V Weiss P Kappler J Voges J Koulousakis A. Bilateral high-frequency stimulation of the internal globus pallidus in advanced Parkinson's disease. Ann Neurol. (1998) 44:953–61. 10.1002/ana.410440615 9851441

[253] Krack P Batir A Van Blercom N Chabardes S Fraix V Ardouin C. Five-year follow-up of bilateral stimulation of the subthalamic nucleus in advanced Parkinson's disease. N Engl J Med. (2003) 349:1925–34. 10.1056/NEJMoa035275 14614167

[254] Williams A Gill S Varma T Jenkinson C Quinn N Mitchell R. Deep brain stimulation plus best medical therapy versus best medical therapy alone for advanced Parkinson's disease (PD SURG trial): a randomised, open-label trial. Lancet Neurol. (2010) 9:581–91. 10.1016/S1474-4422(10)70093-4 20434403 PMC2874872

[255] Castrioto A. Ten-year outcome of subthalamic stimulation in Parkinson's disease: a blinded evaluation. Arch Neurol. (2011) 68:1550. 10.1001/archneurol.2011.182 21825213

[256] Smeding HMM Speelman JD Koning-Haanstra M Schuurman PR Nijssen P van Laar T. Neuropsychological effects of bilateral STN stimulation in Parkinson disease: a controlled study. Neurology. (2006) 66:1830–6. 10.1212/01.wnl.0000234881.77830.66 16801645

[257] Smeding HMM Speelman JD Huizenga HM Schuurman PR Schmand B. Predictors of cognitive and psychosocial outcome after STN DBS in Parkinson's Disease. J Neurol Neurosurg Psychiatry. (2011) 82:754–60. 10.1136/jnnp.2007.140012 19465417

[258] York MK Dulay M Macias A Levin HS Grossman R Simpson R. Cognitive declines following bilateral subthalamic nucleus deep brain stimulation for the treatment of Parkinson's disease. J Neurol Neurosurg Psychiatry. (2008) 79:789–95. 10.1136/jnnp.2007.118786 17965146

[259] Williams AE Arzola GM Strutt AM Simpson R Jankovic J York MK. Cognitive outcome and reliable change indices two years following bilateral subthalamic nucleus deep brain stimulation. Parkinsonism Relat Disord. (2011) 17:321–7. 10.1016/j.parkreldis.2011.01.011 21316292 PMC3109216

[260] Sáez-Zea C Escamilla-Sevilla F Katati MJ Mínguez-Castellanos A. Cognitive effects of subthalamic nucleus stimulation in Parkinson's disease: a controlled study. Eur Neurol. (2012) 68:361–6. 10.1159/000341380 23095782

[261] Ezzyat Y Kragel JE Burke JF Levy DF Lyalenko A Wanda P. Direct brain stimulation modulates encoding states and memory performance in humans. Curr Biol. (2017) 27:1251–8. 10.1016/j.cub.2017.03.028 28434860 PMC8506915

[262] Fell J Staresina BP Do Lam ATA Widman G Helmstaedter C Elger CE. Memory modulation by weak synchronous deep brain stimulation: a pilot study. Brain Stimul. (2013) 6:270–3. 10.1016/j.brs.2012.08.001 22939277

[263] Miller JP Sweet JA Bailey CM Munyon CN Luders HO Fastenau PS. Visual-spatial memory may be enhanced with theta burst deep brain stimulation of the fornix: a preliminary investigation with four cases. Brain. (2015) 138:1833–42. 10.1093/brain/awv095 26106097

[264] Perrine K Devinsky O Uysal S Luciano DJ Dogali M. Left temporal neocortex mediation of verbal memory: evidence from functional mapping with cortical stimulation. Neurology. (1994) 44:1845. 10.1212/WNL.44.10.1845 7936234

[265] Coleshill SG. Material-specific recognition memory deficits elicited by unilateral hippocampal electrical stimulation. J Neurosci. (2004) 24:1612–6. 10.1523/JNEUROSCI.4352-03.2004 14973245 PMC6730466

[266] Lee DJ Izadi A Melnik M Seidl S Echeverri A Shahlaie K. Stimulation of the medial septum improves performance in spatial learning following pilocarpine-induced status epilepticus. Epilepsy Res. (2017) 130:53–63. 10.1016/j.eplepsyres.2017.01.005 28152425

[267] Izadi A Pevzner A Lee DJ Ekstrom AD Shahlaie K Gurkoff GG. Medial septal stimulation increases seizure threshold and improves cognition in epileptic rats. Brain Stimul. (2019) 12:735–42. 10.1016/j.brs.2019.01.005 30733144

[268] Lee DJ Gurkoff GG Izadi A Seidl SE Echeverri A Melnik M. Septohippocampal neuromodulation improves cognition after traumatic brain injury. J Neurotrauma. (2015) 32:1822–32. 10.1089/neu.2014.3744 26096267 PMC4702430

[269] Southwell DG Seifikar H Malik R Lavi K Vogt D Rubenstein JL. Interneuron transplantation rescues social behavior deficits without restoring wild-type physiology in a mouse model of autism with excessive synaptic inhibition. J Neurosci. (2020) 40:2215–27. 10.1523/JNEUROSCI.1063-19.2019 31988060 PMC7083289

[270] Hunt RF Girskis KM Rubenstein JL Alvarez-Buylla A Baraban SC. GABA progenitors grafted into the adult epileptic brain control seizures and abnormal behavior. Nat Neurosci. (2013) 16:692–7. 10.1038/nn.3392 23644485 PMC3665733

[271] Handreck A Backofen-Wehrhahn B Bröer S Löscher W Gernert M. Anticonvulsant effects by bilateral and unilateral transplantation of GABA-producing cells into the subthalamic nucleus in an acute seizure model. Cell Transplant. (2014) 23:111–32. 10.3727/096368912X658944 23191981

[272] Henderson KW Gupta J Tagliatela S Litvina E Zheng X Van Zandt MA. Long-term seizure suppression and optogenetic analyses of synaptic connectivity in epileptic mice with hippocampal grafts of GABAergic interneurons. J Neurosci. (2014) 34:13492–504. 10.1523/JNEUROSCI.0005-14.2014 25274826 PMC4180479

[273] Hammad M Schmidt SL Zhang X Bray R Frohlich F Ghashghaei HT. Transplantation of GABAergic interneurons into the neonatal primary visual cortex reduces absence seizures in stargazer mice. Cerebr Cortex. (2015) 25:2970–9. 10.1093/cercor/bhu094 24812085 PMC4537440

[274] Cunningham M Cho J-H Leung A Savvidis G Ahn S Moon M. hPSC-derived maturing GABAergic interneurons ameliorate seizures and abnormal behavior in epileptic mice. Cell Stem Cell. (2014) 15:559–73. 10.1016/j.stem.2014.10.006 25517465 PMC4270101

[275] Hunt RF Baraban SC. Interneuron transplantation as a treatment for epilepsy. Cold Spring Harb Perspect Med. (2015) 5:a022376. 10.1101/cshperspect.a022376 26627452 PMC4665034

[276] Upadhya D Hattiangady B Castro OW Shuai B Kodali M Attaluri S. Human induced pluripotent stem cell-derived MGE cell grafting after status epilepticus attenuates chronic epilepsy and comorbidities via synaptic integration. Proc Nat Acad Sci USA. (2019) 116:287–96. 10.1073/pnas.1814185115 30559206 PMC6320542

[277] Anderson NC Van Zandt MA Shrestha S Lawrence DB Gupta J Chen CY. Pluripotent stem cell-derived interneuron progenitors mature and restore memory deficits but do not suppress seizures in the epileptic mouse brain. Stem Cell Res. (2018) 33:83–94. 10.1016/j.scr.2018.10.007 30340090

[278] Zhu B Eom J Hunt RF. Transplanted interneurons improve memory precision after traumatic brain injury. Nat Commun. (2019) 10:5156. 10.1038/s41467-019-13170-w 31727894 PMC6856380

